# The Fluorescent Sensing of BF_3_ and Amines: A Dual Approach with Hydrazone Ligands

**DOI:** 10.3390/s24237415

**Published:** 2024-11-21

**Authors:** Haichao Ye, Liqin Liu, Dagang Shen, Chang Song, Huanhuan Wang

**Affiliations:** College of Chemistry and Chemical Engineering, Xinjiang Agricultural University, Urumqi 830002, China

**Keywords:** BF_3_ detection, paper strips, amines, boron difluoride hydrazone, sensor array, principal component analysis, hierarchical clustering analysis

## Abstract

BF_3_, volatile amines (VOAs), and biogenic amines (BAs) are the key indicators in chemical reaction catalysis and food quality monitoring. In this study, we present two types of fluorescent sensors, a hydrazone ligand (HL)-based fluorescent sensor for BF_3_ detection and a novel sensor array using six boron difluoride (BF_2_) hydrazone complexes (BFHs) for monitoring VOAs and BAs. Spectral research indicates that the interaction mechanism between the HLs and BF_3_ is based on intramolecular charge transfer (ICT). The HLs for the monitoring of BF_3_ showed good sensitivity, selectivity, and anti-interference and have the characteristics of a visible color change. Additionally, the HL probe demonstrates reversibility in the presence of triethylamine, making it a candidate for “ON-OFF-ON” mode sensing. BF_3_ detection can also be efficiently performed using test strips for convenient, air-based applications. The BFH sensor array successfully differentiates histamine from the other typical non-volatile BAs in solution; in comparison, the VOAs are analyzed through recognition patterns and statistical analysis. The array’s color changes enable the practical, on-site detection of shrimp spoilage, with principal component analysis distinguishing various ageing intervals. In summary, this sensor array demonstrates high selectivity for VOAs and BAs, with significant potential for application in real-world sample analysis.

## 1. Introduction

Boron trifluoride (BF_3_) is essential in various organic synthesis reactions, including isomerization, condensation, and ionic polymerization, largely due to its high catalytic efficiency [1]. However, BF_3_ is extremely toxic and corrosive; even trace amounts pose serious environmental and health risks due to its strong reactivity with metals and organic compounds [2]. Contact with water can lead to explosions, producing hydrofluoric acid (HF), which is highly hazardous to human health, affecting in particular the skin, eyes, respiratory system, and nasal cavity. Due to the above dangers [3,4], many countries have restricted or banned the use of BF_3_, emphasizing the urgent need for efficient sensors to detect BF_3_ and prevent environmental contamination, production accidents, and gas leakage. In parallel, typical biogenic amines (BAs), such as histamine, tyramine, and putrescine, play key roles in several processes, from neurotransmission to food spoilage [5]. These amines are typically formed by amino acid decarboxylation or through the amination and transamination of aldehydes and ketones [6,7]. While decarboxylation produces non-volatile biogenic amines, amination and transamination lead to the formation of volatile aliphatic amines. In food and beverages, elevated levels of amines—often caused by microbial decarboxylation—pose health risks if not adequately controlled [8,9]. Therefore, understanding their occurrence, physiological and toxicological effects, formation mechanisms, control factors, and methods for on-site ultra-trace detection have become focal areas in food safety research.

However, in recent years, the monitoring of volatile organic amines (VOAs) has also aroused significant interest due to environmental and public safety concerns arising from exposure to these substances [10]. Therefore, a great number of researchers are committed to developing VOA sensors. Once sensing materials are exposed to VOAs, changes in their electrochemical properties, fluorescence, and color provide signals for the detection and identification of analytes [11]. Among the various output signals, the colorimetric mode is considered the most convenient sensing platform for developing simple, visible-to-the-naked-eye VOA detectors because it minimizes the need for complex signal transduction hardware. This advantage promotes practical on-site analysis and can be directly used by non-technical personnel or end users. The currently reported colorimetric sensing materials for VOA sensors include small-molecule organic compounds, metal complexes, and conjugated polymers [12,13]. Over the last decade, traditional analytical techniques, such as high-performance liquid chromatography (HPLC), gas chromatography (GC), and capillary electrophoresis, while effective, have become costly and complex and require skilled operators. These limitations have inspired the development of innovative, fast, and sensitive detection platforms that can be more effectively applied to the detection of toxic and hazardous substances and food [14]. In comparison, UV–Vis absorption and fluorescence techniques offer advantages such as speed, ease of operation, low cost, selectivity, and real-time monitoring, making them valuable tools for detecting and imaging trace analytes [15]. However, successful BF_3_ detection remains rare, with most chemical sensor probes exhibiting drawbacks such as long response times, irreversibility, or a lack of fluorescence response. Concurrently, biogenic amines (BAs) and volatile organic amines (VOAs) are the key indicators of food freshness [16,17] and are prevalent in a wide range of food and environmental contaminants; thus, we must ensure natural environment protection and public safety. Qualitative and quantitative analyses of BAs in spoilage processes [18], as well as VOAs exposed to air [19,20], are particularly common in protein-rich foods [21,22], vegetables, and industrial production and have been widely performed. With the increasing demand for efficient and reliable BA and VOA detection, traditional analytical methods [23,24], while effective, are often costly [25], complex, and require skilled operators [26]. These constraints have driven the development of innovative, fast, and sensitive detection platforms suitable for deployment at all stages of the food supply chain and industrial production [14].

Recent advances have promoted the development of innovative fluorescent and colorimetric biosensors, which offer promising solutions for detecting BF_3_, biogenic amines (BAs), and volatile organic amines [27,28]. Optical analytical technologies have emerged that can detect BF_3_ [29], amines, and food spoilage by monitoring the changes in wavelength or intensity. Notably, visible color changes enable BF_3_ and amines to be directly identified with the naked eye [30]. Moreover, amine detection can now be accomplished using smartphone apps. Therefore, the visual indicators of BF_3_, gaseous amines, and food spoilage are important for on-site trace-level detection [31,32].

Despite recent progress, the design of biosensors for BF_3_, BA, and VOA detection remains relatively underexplored. Developing these technologies requires balancing sensitivity, selectivity, and ease of use to enable rapid and accurate detection of BF_3_, BAs, and VOAs at low concentrations. Additionally, the challenge of distinguishing BF_3_, BAs, and VOAs from structurally similar compounds in complex matrices necessitates the use of sensor arrays and advanced detection methodologies. Most fluorescent probes rely on reactions, such as Knoevenagel and amide reactions, and exhibit pH sensitivity. In this study, two types of fluorescent sensors were developed, a fluorescent sensor using hydrazone ligands (HLs) coordinated with boron trifluoride for BF_3_ detection and a novel fluorescent sensor array composed of six boron difluoride (BF_2_) complexes (BFHs) for monitoring volatile amines (VOAs) and biogenic amines (BAs). Multivariate analysis, including hierarchical cluster analysis (HCA) and principal component analysis (PCA), was employed to differentiate the histamines, the volatile amines, and the spoilage stages in shrimp, enabling practical on-site monitoring applications.

## 2. Experimental Section

### 2.1. Materials and Instruments

Phosphorus tribromide, triethyl phosphite, methyl 6-(hydroxymethyl)picolinate, 4-diphenylamino benzaldehyde, carbazole, 3,6-di-tert-butyl carbazole, 4-fluorobenzaldehyde, phenoxazine, phenothiazine, p-dimethylaminobenzaldehyde, phenyl hydrazine, diisobutyl aluminum hydride (DIBAL-H), potassium tert-butoxide, boron trifluoride ethyl ether, potassium carbonate, potassium phosphate and potassium tert-butoxide were purchased from Yinokai Reagent (Beijing, China). Histamine, putrescine, tyramine, tryptamine, 2-phenylethylamine, spermidine, spermine, and cadaverine were purchased from Aladdin Biochemical Technology (Shanghai, China). Ammonia, methylamine, ethylamine, dimethylamine, diethylamine, trimethylamine, triethylamine, propylamine and butylamine were purchased from McLean Reagent (Shanghai, China). All reagents were used without further purification. Mass spectra (ESI) were acquired using a Bruker MicroTOF ESI-TOF Mass Spectrometer (Billerica, MA, USA). ^19^F NMR, ^1^H NMR, and ^13^C NMR spectra were recorded on a JNM-ECS-400 MHz spectrometer (JEOL, Tokyo, Japan). Fluorescence spectra were obtained using an Agilent Cary Eclipse G9800A spectro fluorophotometer (Santa Clara, CA, USA) with both excitation and emission slit widths set to 5.0 nm. The UV-vis spectra were measured on a Shimadzu UV2600 Series UV–vis spectrophotometer (Kyoto, Japan).

### 2.2. Sensitivity and Selectivity Studies

In order to perform UV–visible absorption and emission titration experiments, we prepared stock solutions of the probe HLs (10 μM) by dissolving them in solvents of spectroscopic grade of THF/H_2_O (9:1, *v*/*v*) media. The nitrate salts (1 mM) of various metals including Pd^3+^, Ni^2+^, Fe^3+^, Cr^3+^, Co^2+^, Zn^2+^, Ca^2+^, Mg^2+^, Al^3+^, Cu^2+^, Cd^2+^, Hg^2+^, Ag^+^, and other boron species (1 mM) such as BBr_3_, B (OMe)_3_, BPh_3_, H_3_BO_3_, BCl_3_, NaBF_4_, Borax, and BF_3_ were dissolved in double-distilled water.

### 2.3. Limit of Detection (LOD)

To calculate the LOD, the expression LOD = 3SD/slope was employed. In this expression, SD stands for the standard deviation obtained from analyzing ten replicates of the HLs. A slope can be seen from the calibration graph of emission intensity versus BF_3_ concentration.

### 2.4. Preparation of Paper Strips

A filter paper strip was immersed in a dichloromethane solution of sensor HL (1 mM) and then air-dried for 20 min. Subsequently, these coated paper strips were exposed to varying concentrations of BF_3_, prepared by diluting a stock solution (1 × 10^−3^ M) to final concentrations of 20 μM, 40 μM, 60 μM, 80 μM, 100 μM, 200 μM, 300 μM, and 500 μM. Under 365 nm UV light, the color of the test paper changed from colorless to various fluorescent hues.

### 2.5. Fabrication of the Fluorescent Sensor Array

The fluorescent sensor array was prepared by immersing the filter paper in dye solutions. First, the HL probes were each dissolved in 100 mL of CH_2_Cl_2_ and subsequently mixed with BF_3_·Et_2_O in a 1:1 ratio to produce 2 mM dye solutions. Small filter paper strips (1 × 3 cm) were then soaked in these dye solutions for 1 min, placed on a slide, and allowed to dry at room temperature. The resulting six sensors were carefully arranged to assemble the fluorescent sensor array.

### 2.6. Acquisition of Images of the Fluorescent Sensor Arrays

A dark cardboard box platform, integrated with a smartphone and a 365 nm UV light, was used to capture images of the fluorescent sensor array. The smartphone was fixed at a vertical distance of 300 mm above the base of the box to standardize the photography conditions. The RGB values for each sensor’s color were obtained using the color picker tool (5 × 5 average sample size) in Adobe Photoshop CC software (2019 release, Adobe Inc., San Jose, CA, USA).

### 2.7. Statistical Analysis

All statistical analyses were conducted using SPSS software (Version 25.0, IBM Inc., New York, NY, USA). Data are presented as the mean ± standard deviation, based on five replicates.

### 2.8. General Procedures for Hydrazone Compounds

All molecular synthetic routes are shown in Scheme S1, and the specific synthetic methods are given below:

9-(4-((E)-2-(6-((Z)-(2-phenylhydrazineylidene)methyl)pyridin-2-l) vinyl)phenyl)-9H-carbazole (ENN). A solution of (E)-9-(4-(2-(6-methylpyridin-2-yl)vinyl)phenyl)-9H-carbazole (0.10 g, 0.22 mmol) dissolved in 10 mL methanol and a few drops of acetic acid were added to phenylhydrazine (150 µL, 1.38 mmol). The solution was stirred at 70 °C for 2 h. The solution was cooled to room temperature, and the yellow solids were filtered and collected. Yield 86%. ^1^H NMR (400 MHz, DMSO) δ 10.69 (s, 1H), 8.23 (dt, J = 7.8, 1.0 Hz, 2H), 7.99–7.92 (m, 3H), 7.86–7.73 (m, 3H), 7.68–7.61 (m, 2H), 7.47 (dd, J = 7.4, 1.3 Hz, 1H), 7.44–7.37 (m, 5H), 7.33–7.20 (m, 4H), 7.15–7.08 (m, 2H), 6.79 (tt, J = 7.3, 1.1 Hz, 1H). ^13^C NMR (101 MHz, CDCl_3_) δ 154.66, 154.50, 144.00, 140.67, 140.64, 137.53, 137.38, 136.83, 135.73, 131.76, 129.34, 128.44, 127.12, 125.99, 123.47, 120.93, 120.76, 120.33, 120.07, 118.35, 113.01, 112.99, 109.85. HRMS (ESI^+^) calcd for C_32_H_24_N_4_ [M + H]^+^: 465.2072, found 465.2071 (Figures S1–S3).

3,6-di-tert-butyl-9-(4-((E)-2-(6-((Z)-(2-phenylhydrazineylidene)methyl)pyridin-2-yl)vinyl)phenyl)-9H-carbazole (TNN). ^1^H NMR (400 MHz, DMSO) δ 10.68 (s, 1H), 8.26 (d, J = 2.0 Hz, 2H), 7.95–7.88 (m, 3H), 7.84–7.71 (m, 3H), 7.64–7.57 (m, 2H), 7.48–7.40 (m, 4H), 7.34 (t, J = 8.4 Hz, 3H), 7.26–7.20 (m, 2H), 7.14–7.07 (m, 2H), 6.78 (tt, J = 7.2, 1.2 Hz, 1H), 1.38 (s, 18H). ^13^C NMR (101 MHz, DMSO) δ 155.10, 154.87, 145.12, 143.09, 138.77, 137.60, 137.50, 137.27, 135.55, 131.48, 129.66, 129.05, 126.80, 124.18, 123.40, 121.51, 119.95, 117.85, 117.14, 112.75, 109.61, 34.94, 32.26. HRMS (ESI^+^) calcd for C_40_H_40_N_4_ [M + H]^+^: 577.3325, found 577.3329 (Figures S4–S6).

N,N-diphenyl-4-((E)-2-(6-((Z)-(2-phenylhydrazineylidene)methyl)pyridin-2-yl)vinyl)aniline (FNN). ^1^H NMR (400 MHz, DMSO) δ 10.64 (s, 1H), 7.88 (s, 1H), 7.78–7.67 (m, 2H), 7.61–7.52 (m, 3H), 7.39–7.27 (m, 5H), 7.22 (t, J = 7.7 Hz, 2H), 7.17–7.00 (m, 9H), 6.92 (d, J = 8.4 Hz, 2H), 6.77 (t, J = 7.3 Hz, 1H).^13^C NMR (101 MHz, CDCl3) δ 148.59, 147.18, 143.65, 138.10, 134.47, 129.63, 129.39, 129.35, 129.31, 128.42, 128.30, 128.18, 125.04, 124.97, 124.91, 124.86, 123.63, 123.52, 123.40, 122.50, 121.12, 119.75, 117.64, 113.21.HRMS (ESI^+^) calcd for C_32_H_26_N_4_ [M + H]^+^: 467.2236, found 467.2221 (Figures S7–S9).

N,N-dimethyl-4-((E)-2-(6-((Z)-(2-phenylhydrazineylidene)methyl)pyridin-2-yl)vinyl)aniline (LNN). ^1^H NMR (400 MHz, DMSO) δ 10.62 (s, 1H), 7.87 (s, 1H), 7.78–7.59 (m, 2H), 7.58–7.43 (m, 3H), 7.31 (dd, J = 6.6, 2.0 Hz, 1H), 7.22 (dd, J = 8.5, 7.2 Hz, 2H), 7.15–7.04 (m, 2H), 6.99 (d, J = 16.0 Hz, 1H), 6.82–6.67 (m, 3H), 2.92 (s, 6H).^13^C NMR (101 MHz, CDCl_3_) δ 155.79, 153.93, 150.64, 144.07, 137.54, 136.79, 133.43, 129.34, 128.48, 124.79, 122.87, 120.70, 119.96, 117.16, 113.02, 112.23, 77.23, 40.36.HRMS (ESI^+^) calcd for C_22_H_22_N_4_ [M + H]^+^: 343.1829, found 343.1911 (Figures S10–S12).

10-(4-((E)-2-(6-((Z)-(2-phenylhydrazineylidene)methyl)pyridin-2-yl)vinyl)phenyl)-10H-phenothiazine (PNN). ^1^H NMR (400 MHz, DMSO) δ 10.67 (s, 1H), 7.93–7.86 (m, 3H), 7.84–7.67 (m, 3H), 7.43 (d, J = 7.3 Hz, 1H), 7.40–7.31 (m, 3H), 7.22 (t, J = 7.7 Hz, 2H), 7.13–7.05 (m, 4H), 6.95 (td, J = 7.8, 1.6 Hz, 2H), 6.86 (td, J = 7.5, 1.2 Hz, 2H), 6.77 (t, J = 7.3 Hz, 1H), 6.30 (dd, J = 8.4, 1.3 Hz, 2H).^13^C NMR (101 MHz, DMSO) δ 155.09, 154.79, 145.11, 143.79, 140.94, 137.50, 137.25, 136.40, 131.34, 130.01, 129.87, 129.66, 129.45, 127.82, 127.28, 123.49, 121.54, 120.87, 119.95, 117.91, 117.35, 112.75.HRMS (ESI^+^) calcd for C_32_H_24_N_4_S [M + H]^+^: 497.1776, found 497.1784 (Figures S13–S15).

10-(4-((E)-2-(6-((Z)-(2-phenylhydrazineylidene)methyl)pyridin-2-yl)vinyl)phenyl)-10H-phenoxazine (ONN). ^1^H NMR (400 MHz, DMSO) δ 10.68 (s, 1H), 7.97–7.90 (m, 3H), 7.82–7.69 (m, 3H), 7.48–7.35 (m, 4H), 7.23 (t, J = 7.8 Hz, 2H), 7.11 (d, J = 8.0 Hz, 2H), 6.79 (t, J = 7.3 Hz, 1H), 6.75–6.66 (m, 3H), 6.64 (dd, J = 4.5, 2.4 Hz, 3H), 5.94–5.86 (m, 2H). ^13^C NMR (101 MHz, DMSO) δ 155.11, 154.72, 145.10, 143.59, 138.48, 137.52, 137.26, 137.22, 134.22, 131.28, 131.22, 130.23, 129.81, 129.66, 124.18, 121.94, 121.61, 119.96, 117.98, 115.76, 113.65, 112.75. HRMS (ESI^+^) calcd for C_32_H_24_N_4_O [M + H]^+^: 481.2042 found 481.20118 (Figures S16–S18).

## 3. Results and Discussion

### 3.1. Principles of Design

**Figure 1:**
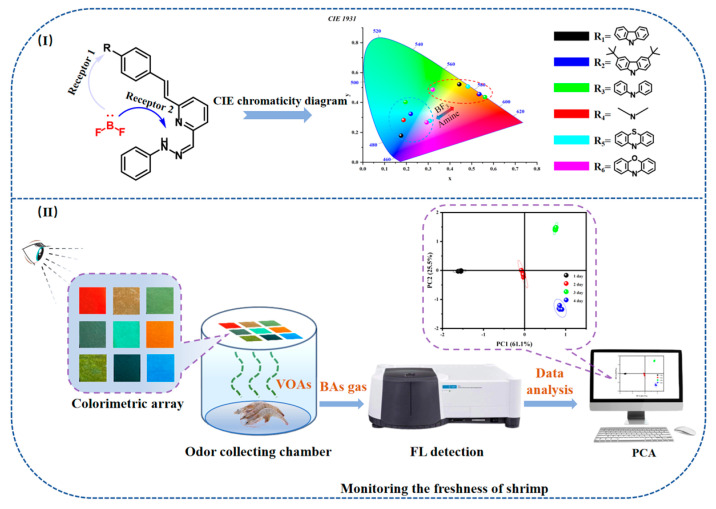
(**I**) The design principles of sensors; (**II**) The detection and analysis of amine by fluorescence sensor array.

In this study, we synthesized six hydrazone-based ligands with features of internal charge transfer (ICT). These molecular scaffolds incorporate hydrazone components functioning as electron acceptors and nitrogenous groups serving as electron donors. The optical properties of these ligands can be modulated due to the varying electron-donating capabilities of the incorporated donor groups, thereby enabling the development of a multi-color luminescence platform. Furthermore, the hydrazone moieties and nitrogenous functionalities are capable of coordinating with BF_3_, a process that can be influenced by the presence of alkaline substances. As a result, each scaffold possesses two receptor sites for amines. The CIE diagram clearly shows that the fluorescence color of each sensor differs significantly before and after exposure to BF_3_ and amines. This observation can be utilized in the design and development of detectors for BF_3_ and amines. Therefore, by developing a hydrazone ligand (HL)-based fluorescent sensor for BF_3_ detection and capitalizing on its multi-color and multi-receptor characteristics, we envision the fabrication of a sensor array utilizing these molecules as components. This array can detect biogenic amines in solution and volatile amines in vapor via multivariate analysis. Additionally, this system has been practically implemented to monitor food freshness through visual assessment and statistical data analysis, as shown in Figure 1.

### 3.2. UV–Vis Spectra Response to BF_3_

**Figure 2:**
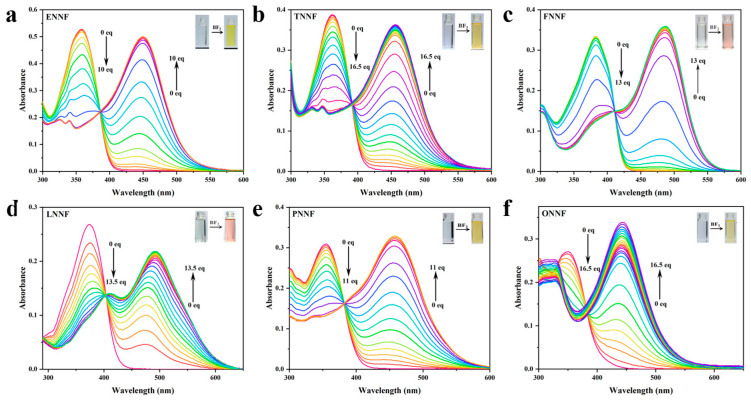
(**a**–**f**) UV–vis absorption spectra of HLs (5 μM) in the presence of BF_3_ at different concentrations (0–16.5 equivalents) in CH_2_Cl_2_.

To evaluate the feasibility of six HLs for BF_3_ detection, the UV–vis absorption spectra of the HLs were examined in CH_2_Cl_2_ at room temperature with increasing concentrations of BF_3_ [33]. As depicted in Figure 2a–f, the HL probes initially exhibited maximum absorption peaks at 358 nm, 362 nm, 382 nm, 375 nm, 355 nm, and 350 nm, respectively. Upon the addition of BF_3_, these peaks underwent a significant enhancement and red-shifted to 450 nm, 457 nm, 487 nm, 492 nm, 458 nm, and 443 nm, respectively. This spectral shift was accompanied by a distinct color change in the probe solutions, transitioning from colorless to light yellow or pink. With the further addition of BF_3_, the maximum absorption peaks of the six HLs in Figure 2a–f exhibited red shifts of 92 nm, 95 nm, 104 nm, 119 nm, 105 nm, and 93 nm, respectively, accompanied by a noticeable color change in the probe solution from colorless to pale yellow or pink. This result indicates that HL probes serve as effective indicators for “naked eye” BF_3_ detection.

### 3.3. Colorimetric and Fluorometric Titration Studies of HLs

**Figure 3:**
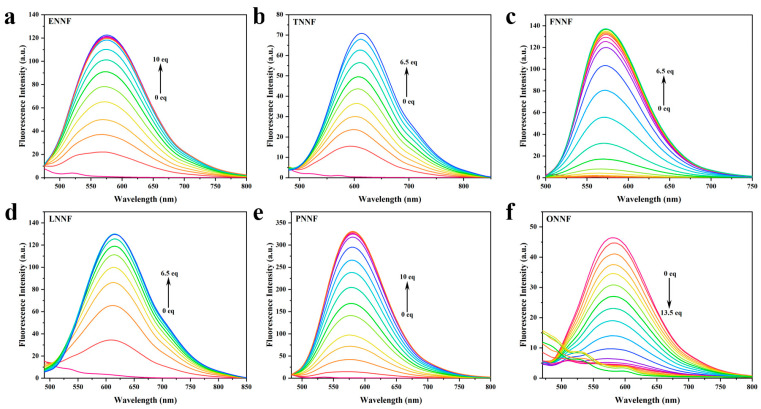
(**a**–**f**) Fluorescence spectra of HLs (5 μM) with the addition of BF_3_ at different concentrations (0–13.5 equivalents) in CH_2_Cl_2_. The excitation wavelength was 460 nm.

**Figure 4:**
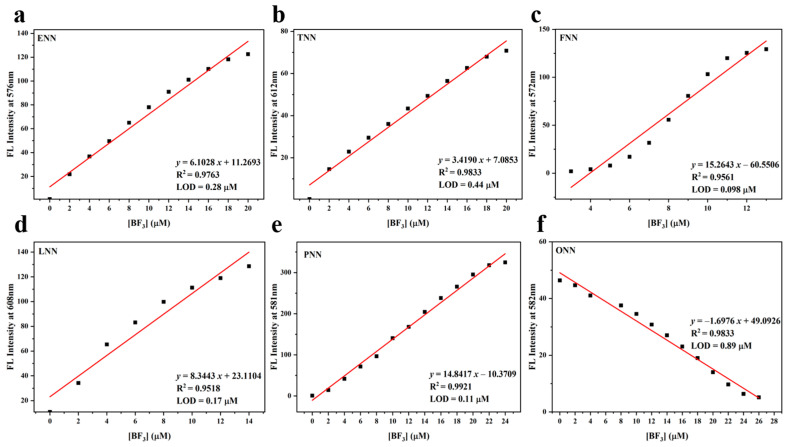
(**a**–**f**) The linear fitting relationships of the concentration of BF_3_ against the fluorescence intensity for HLs.

To investigate the quantitative response of six HLs to various BF_3_ concentrations, fluorescence titration experiments were conducted in CH_2_Cl_2_ at room temperature [34,35]. Using dual excitation wavelengths at 460 nm and 365 nm, as shown in Figure 3, the fluorescence intensity of the HLs between 550 and 650 nm gradually increased with rising BF_3_ concentration, reaching a plateau between 6.5 and 13.5 equivalents of BF_3_. As illustrated in Figure S23, with increasing BF_3_ addition, the fluorescence intensity between 425 and 600 nm gradually decreased, stabilizing at a plateau between 6.5 and 13.5 equivalents of BF_3_. Simultaneously, the fluorescence color shifted from chartreuse to bright red. These observations, attributed to an intramolecular charge transfer (ICT) mechanism within the BFH molecules formed by the reaction of the HL ligands with BF_3_, demonstrate that the HL probes enable highly sensitive, quantitative detection of BF_3_. Furthermore, based on the linear fitting relationship of fluorescence intensity against the titrated concentrations of BF_3_ in Figure 4a–f, the limits of detection (LODs) of ENN, TNN, FNN, LNN, PNN, and ONN were calculated to be 0.28 μM, 0.44 μM, 0.098 μM, 0.17 μM, 0.11 μM, and 0.89 μM, respectively. As demonstrated in our study, the proposed HL probes provide more sensitive detection capabilities and a “turn-on”-type probe.

### 3.4. Selectivity Studies

**Figure 5:**
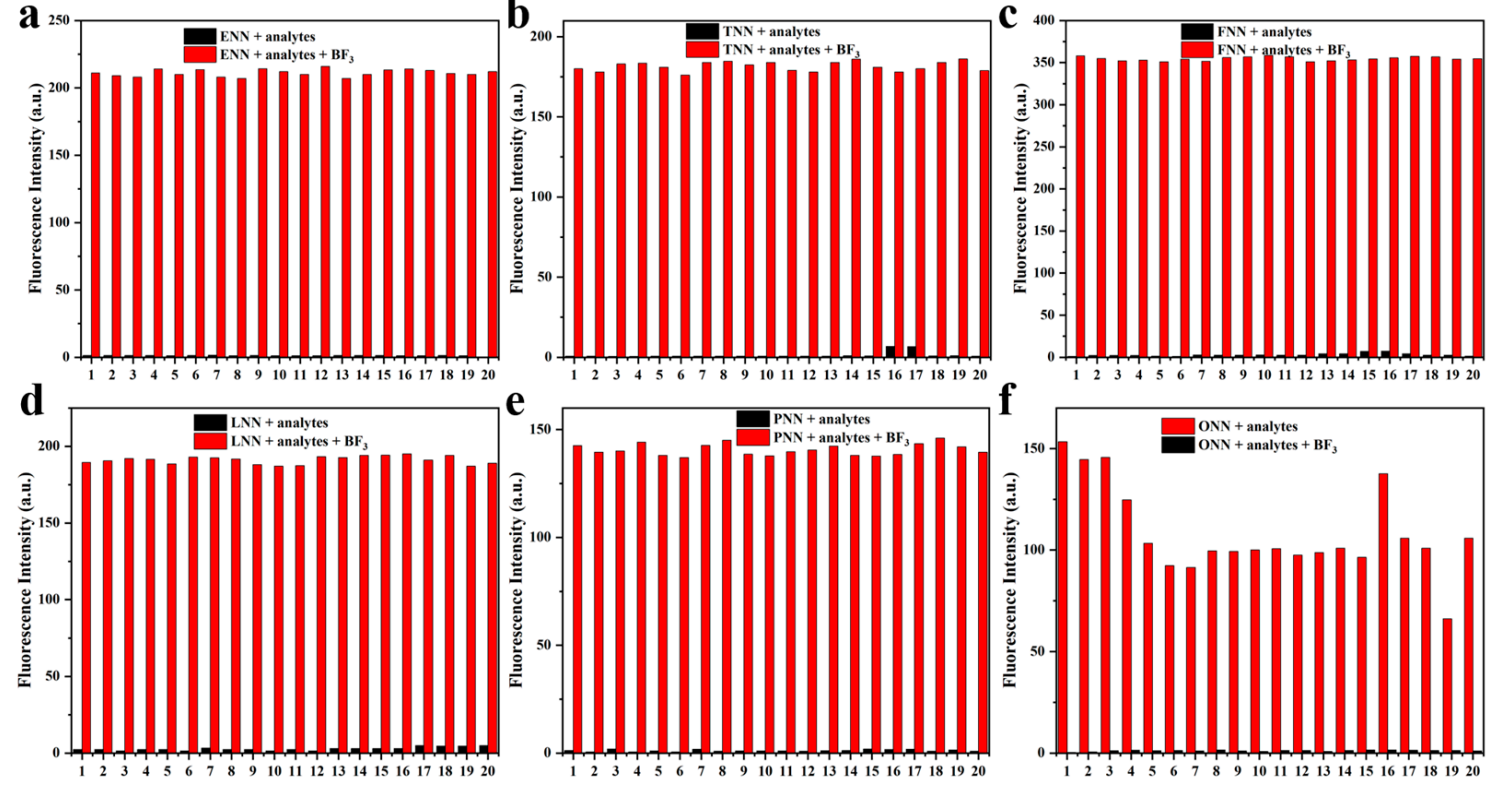
(**a**–**f**) Fluorescence intensity spectra of HLs and the HLs + BF_3_ complex with various competitive species. 1: Pd^3+^, 2: Ni^2+^, 3: Fe^3+^, 4: Cr^3+^, 5: Co^2+^, 6: Zn^2+^, 7: Ca^2+^, 8: Mg^2+^, 9: Al^3+^, 10: Cu^2+^, 11: Cd^2+^, 12: Hg^2+^, 13: Ag^+^, 14: BBr_3_, 15: B(OMe)_3_, 16: BPh_3_, 17: H_3_BO_3_, 18: BCl_3_, 19: NaBF_4_, 20: Borax, and 21: BF_3_.

To evaluate the sensitivity and selectivity of the probe HLs for boron species and metal cations, we employed UV–vis absorption and fluorescence spectroscopy (Figures S25 and S26) in a THF/H_2_O (9:1, *v*/*v*) solution for Pd^3+^, Ni^2^⁺, Fe^3^⁺, Cr^3^⁺, Co^2^⁺, Zn^2^⁺, Ca^2^⁺, Mg^2^⁺, Al^3^⁺, Cu^2^⁺, Cd^2^⁺, Hg^2^⁺, Ag⁺, BBr_3_, B(OMe)_3_, BPh_3_, H_3_BO_3_, BCl_3_, NaBF_4_, Borax, and BF_3_. Figure 5 clearly shows that the HLs + BF_3_ complex with other competitive species did not exhibit any spectral changes in fluorescence intensity, indicating the superior selectivity and good anti-interference of the probe HLs for BF_3_ detection.

### 3.5. Response Time Studies

**Figure 6:**
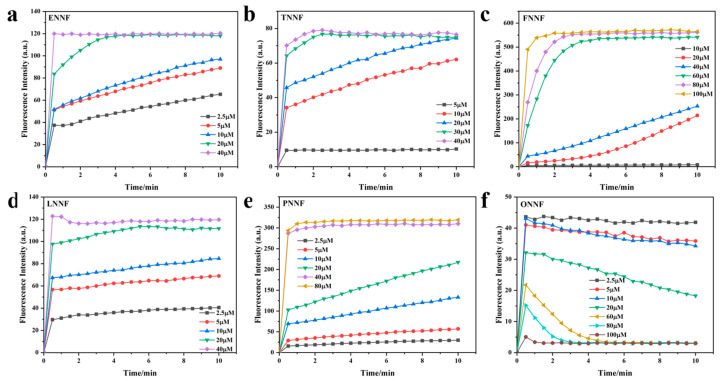
(**a**–**f**) The time-dependent fluorescence intensities changes of 5 μM HLs after the addition of 2.5~100 μM BF_3_ in CH_2_Cl_2_ within 10 min, respectively.

The response time of the probes to BF_3_ was investigated in CH_2_Cl_2_ [33,36]. As shown in Figure 6a–e, the free probes of the five HLs (5 μM) exhibited no changes in fluorescence intensity. However, upon the addition of BF_3_ at concentrations of 40, 30, 100, 40, and 40 μM, respectively, the fluorescence intensity of all five HLs sharply increased, reaching a steady state within 2 min. These results indicate that HLs exhibit a relatively short recognition response time for BF_3_. Conversely, as shown in Figure 6f, when BF_3_ is added at a concentration of 100 μM, the fluorescence intensity of ONN decreases rapidly and reaches a plateau within 2 min. The reason for this phenomenon is the fact that as the BF_3_ concentration increases, the oxygen-based acridine group becomes more likely to combine with BF_3_, reacting via Lewis acid–base interactions to form a complex, rather than coordinating with nitrogen atoms to induce an ICT (intramolecular charge transfer) effect. As a result, there is a gradual decrease in fluorescence intensity. These findings strongly suggest that these ultrafast-response HL probes are suitable for the real-time detection of BF_3_.

### 3.6. Paper Strips Measurement

**Figure 7:**
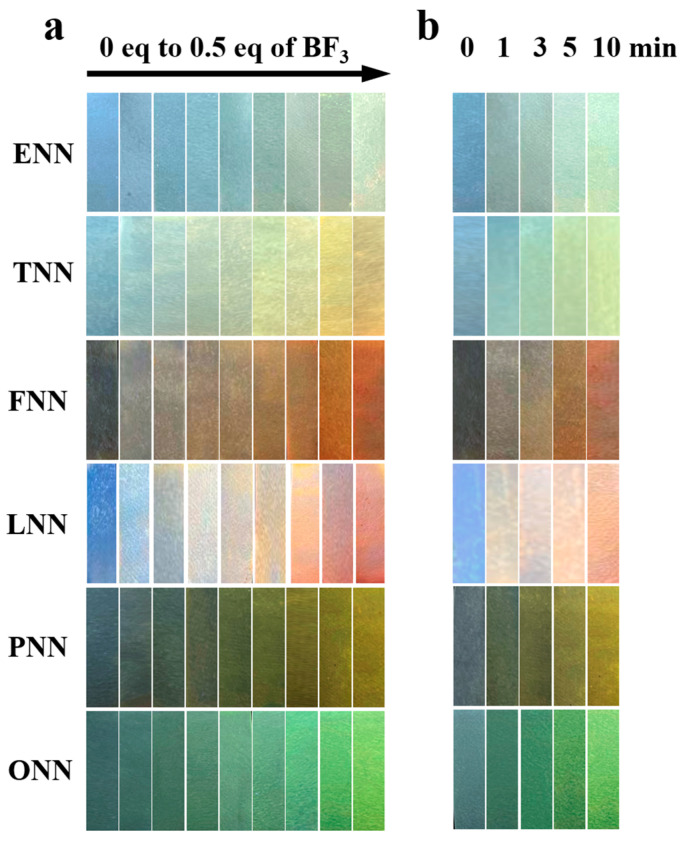
(**a**) Fluorescence photographs of test paper strip containing probe HLs with different concentration of BF_3_ under 365 nm UV light. (**b**) Fluorescence photographs of test papers exposed to BF_3_ (0.2 equivalents) in different time under under 365 nm UV light.

To evaluate the practical application of the six HL probes [37,38], filter paper strips were immersed in a CH_2_Cl_2_ solution of the probes and then dried in a low-temperature oven for 20 min. Thereafter, the coated strips were fumigated in a confined space with a BF_3_ vapor concentration of 0 to 0.5 equivalents for 20 min. As shown in Figure 7a, upon UV exposure, the HL strips displayed distinct fluorescence color changes, indicating that the paper strip method effectively facilitated complex formation with BF_3_ in CH_2_Cl_2_. In a separate test, the same paper strips were exposed to BF_3_ vapor, and as seen in Figure 7b, they exhibited increasing fluorescence intensity over time under UV light. These results suggest that the HL probe-coated paper strips provide a cost-effective, solid-state method for BF_3_ detection, demonstrating excellent fluorescence response. This approach provides a practical, turn-on colorimetric fluorescent sensor suitable for “in-field” BF_3_ measurement.

### 3.7. Screening of HLs

BF_3_ readily forms borate complexes with electron-donating atoms. The electron-donating capacity and the number of atoms in the coordinating ring systems are key factors influencing borate complex formation. Consequently, we selected HLs containing N, N atoms as potential chelators for BF_3_ [38]. Our UV–vis spectral analysis results confirmed that these ligands coordinate with BF_3_. To further investigate the binding mechanism of the six HLs with BF_3_, ^19^F NMR and ^1^H NMR analyses were conducted to examine the binding mode of BF_2_–hydrazone coordination (BFHs), as shown in Figures S19 and S20.

**Figure 8:**
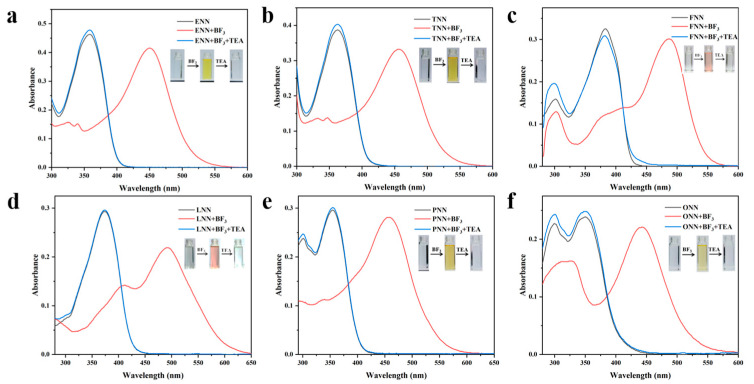
(**a**–**f**) UV-Vis spectra of six hydrazone ligands (5 µM) + BF_3_ (20 equivalents) followed by the addition of Et_3_N (20 equivalents) in CH_2_Cl_2_. Inset: Photographs of color changes under sunlight.

In the absorption spectra, the HLs exhibited bands at λ_abs_ 350–400 nm. Upon the addition of BF_3_, significant, red-shifted absorptions appeared at λ_abs_ 400–500 nm, relative to the free ligands. As shown in the inset of Figure 8, this shift was accompanied by a distinct color change in the solution, from colorless to yellow or red. The effects of the solvent on the UV–vis and fluorescence spectra of the HLs were also investigated, with the results presented in Figures S21 and S22. The results indicate that the HLs exhibited the highest absorption and fluorescence intensities in dichloromethane.

In addition, the emission spectra of the ligands to BF_3_ were also conducted, as shown in Figure S24. Under dual excitation wavelengths at 365 nm and 460 nm, the HLs exhibited sequential and reversible responses to BF_3_ and Et_3_N through two emission channels, respectively. With regard to the response behavior in the short-wavelength channel excited at 365 nm, the six HLs can be categorized into three distinct types. For ligands composed of carbazole (ENN), tert-butyl carbazate (TNN), diphenylamine (FNN), and N, N-dimethylamine groups (LNN), the addition of BF_3_ led to intensity decreases of 21%, 35%, 70%, and 88%, respectively. The ligand incorporating a sulfur-based acridine displayed a hypsochromic shift accompanied by a slight decrease in intensity; in comparison, the ligand containing an oxygen-based acridine showed a ratiometric response. We noted that all spectral changes were reversible upon the addition of Et_3_N.

**Figure 9:**
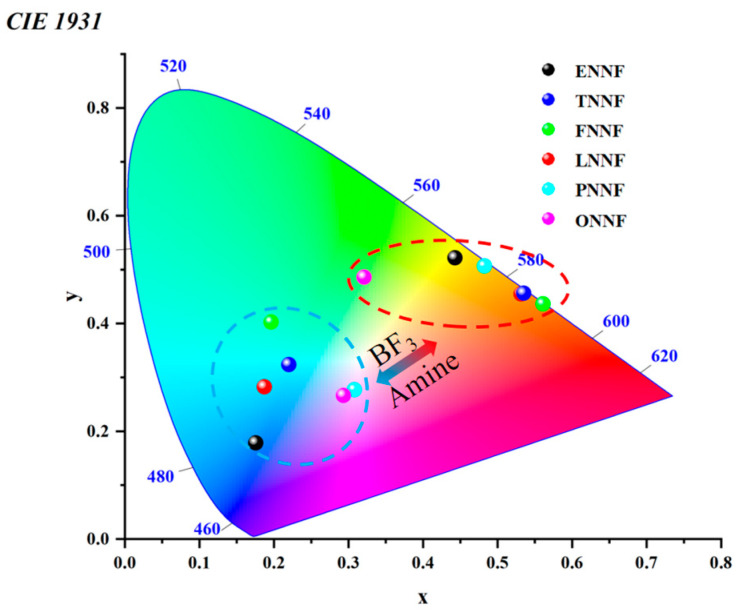
CIE chromaticity diagram of hydrazone ligands to BF_3_ and amines, reversibly.

Based on the response behavior observed in the longer-wavelength channel excited at 460 nm, the six HLs can also be classified into three distinct types. The ligands ENN, TNN, FNN, and LNN exhibited fluorescence switches that were activated upon the addition of BF_3_, resulting in a slight increase in fluorescence intensity to varying degrees. In contrast, the sulfur-based acridine ligand (PNN) demonstrated a reversible fluorescence emission switching phenomenon characterized by a significant increase in intensity, while the oxygen-based acridine ligand (ONN) exhibited the opposite behavior. Notably, with the exception of ONN, all spectral changes were reversible upon the addition of Et_3_N (Figure S24). The apparent difference between the sulfur-based acridine group and the oxygen-based acridine group may be attributed to the fact that BF_3_ acts as a Lewis acid [35]. The sulfur-based acridine group reacts more rapidly than the Lewis basic ligand containing the oxygen-based acridine group within the molecular structure [29]. The reaction products influence the electron-donating properties of the sulfur-based acridine group, hindering the intramolecular charge transfer (ICT) effect and resulting in a blue shift of the spectral peak in the short-wavelength channel. Nonetheless, most BF_3_ tends to form a stable six-membered ring structure through coordination with nitrogen atoms, leading to a significant enhancement in fluorescence intensity. In contrast, the oxygen-based acridine group is more prone to combining with BF_3_, undergoing a Lewis acid–base reaction to form a complex, rather than coordinating with nitrogen atoms, ultimately resulting in fluorescence quenching. Furthermore, Figure 9 illustrates the CIE chromaticity diagram of the BFHs in solution. These BFHs exhibited a chromaticity value that spanned a wide color gamut, ranging from blue to red hues, making them exceptionally ideal for the development of multicolor sensor arrays. These remarkable changes in the UV-vis and fluorescence spectra indicate that BFHs can be used as potential tools for sensitively detecting various amines.

### 3.8. Fluorescence Sensor Array for the Differentiation of Biogenic Amines

**Figure 10:**
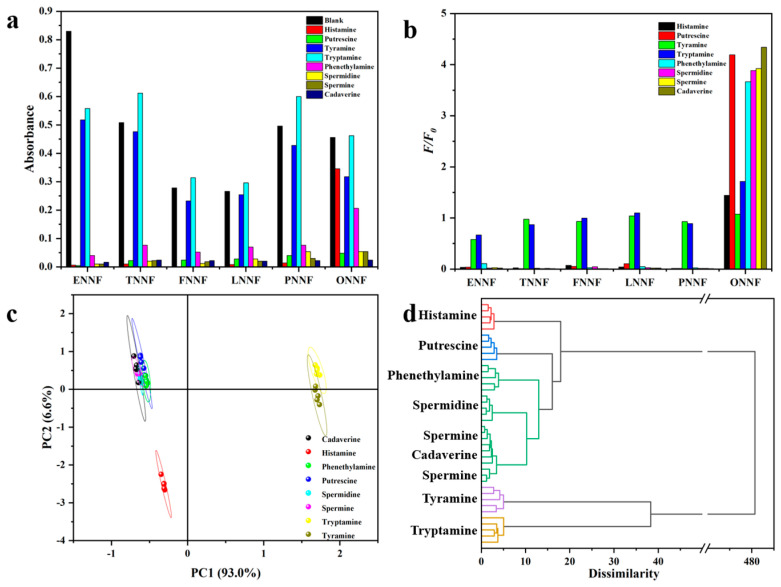
Fluorescence sensor array for the differentiation of biogenic amines. (**a**) UV-vis spectra responses of eight biogenic amines. (**b**) Fluorescence responses of eight biogenic amines. (**c**) PCA score plot of fluorescence intensity changes obtained from the pattern F/F_0_ data (8 amines × 5 replicates). (**d**) Hierarchical cluster analysis (HCA) for sensor array response. All analytes were run in quintuplicate trials.

The UV–vis and fluorescence responses of eight biogenic amines (histamine, putrescine, tyramine, tryptamine, 2-phenylethylamine, spermidine, spermine, and cadaverine) to BFHs were recorded, as shown in Figure 10a,b. When the dyes (5 μM) were mixed with each amine (10 equivalents), the fluorescence intensities of the BFHs at 500–600 nm varied in response (Figure S28). Similarly, the fluorescence response trends were mirrored in the absorption spectra (Figure S27). Specifically, histamine, putrescine, 2-phenylethylamine, spermidine, spermine, and cadaverine markedly quenched BFH fluorescence intensity; in comparison, tyramine and tryptamine had only minor effects on the emission profiles. The resulting fluorescence intensities were used to generate F/F_0_ data patterns, revealing distinct BFH responses to each biogenic amine (Figure 10b). These data were further analyzed using pattern recognition methods to distinguish among the eight amines. Principal Component Analysis (PCA) transformed this fluorescence response patterns (F/F_0_ data) into “fingerprints”, producing two factors: factor 1 (93.0%) and factor 2 (6.6%), which formed a two-dimensional (2D) plot. This plot effectively separated the amines into three clusters (Figure 10c). Notably, histamine was distinctly separated from the other amines, while tyramine and tryptamine showed overlap. Additionally, hierarchical cluster analysis (HCA) was used to assess the comparability among the eight amines. As shown in Figure 10d, all 40 cases (8 amines × 5 replicates) were accurately grouped without misclassification. The combined results from PCA and HCA indicate that this sensor array platform enables rapid, accurate, and reliable identification of biogenic amines, demonstrating strong feasibility and accuracy.

### 3.9. Fluorescence Sensor Array for the Differentiation of Volatile Amines

**Figure 11:**
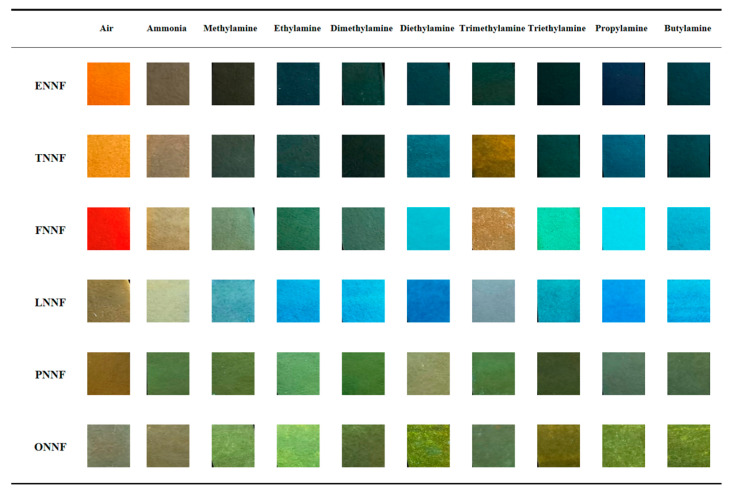
Scanned images of the paper-based sensor array prepared from BFHs exposed to various 10 ppm vapors of VOAs.

**Figure 12:**
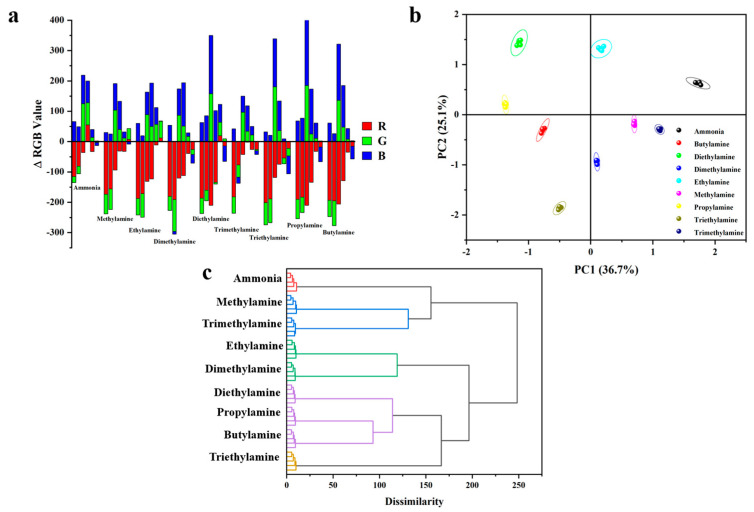
Fluorescence sensor array for the differentiation of volatile amines. (**a**) RGB color change profile of a paper-based sensor array prepared from 6 dyes after exposure to 10 ppm vapors of VOAs. (**b**) PCA score plot of RGB color changes obtained from the paper-based sensor array upon exposure to 9 VOA vapors. (**c**) Hierarchical clustering analysis (HCA) tree of sensor arrays with 6 dyes exposed to 9 VOA vapors. (All experiments were conducted with separate sensor arrays, five times).

To evaluate the applicability of the as-prepared colorimetric sensor array for distinguishing volatile amines, nine different volatile amines were tested at a concentration of 10 ppm. Upon exposure to each amine sample, the array underwent specific reactions, producing distinct color changes displayed in final difference maps (Figure 11). These color change profiles showed unique patterns for each of the nine amines, indicating that the prepared sensor array holds promise for further analytical applications. The color changes were captured using a standard flatbed scanner, and the red, green, and blue (RGB) values for each array element were obtained by subtracting the initial image from the image taken after amine exposure (Figure 12a).

To further assess the sensory performance of the prepared sensor array, statistical methods were applied to analyze the experimental data using hierarchical cluster analysis (HCA) and principal component analysis (PCA), techniques commonly employed in similar studies. In these analyses, variables were defined by the relative changes in each color vector (ΔR^2^, ΔG^2^, or ΔB^2^, divided by the total squared Euclidean distance), totaling 270 parameters (6 dyes × 9 amines × 5 repetitions) for statistical evaluation. Each data point was measured five times with five parallel samples (Figure 12b). The pKa values of the nine volatile amines tested—ammonia, methylamine, ethylamine, dimethylamine, diethylamine, trimethylamine, triethylamine, propylamine, and butylamine—are 9.25, 10.66, 10.71, 10.73, 10.98, 9.74, 11.01, 10.57, and 10.77, respectively. The differences in basicity among these amines result in unique acid–base interactions with the BFH molecules, leading to distinct color changes in the sensor array before and after exposure. Consequently, PCA analysis effectively differentiated these nine amines into distinct clusters, allowing for clear discrimination among them.

Hierarchical cluster analysis (HCA), a multivariate statistical method involving agglomerative and divisive approaches, was used to identify clusters within the dataset. The results clearly indicate that the sensor array demonstrates sufficient selectivity to classify different amines when exposed to VOA vapors. Furthermore, our HCA analysis results indicate the potential for expanding the sensor system’s application, particularly by establishing a comprehensive database of difference maps for various amines (Figure 12c). This database would enhance the system’s effectiveness in accurately distinguishing between amines in future analyses.

### 3.10. The Application of the Sensor Array to Monitor Shrimp Spoilage

**Figure 13:**
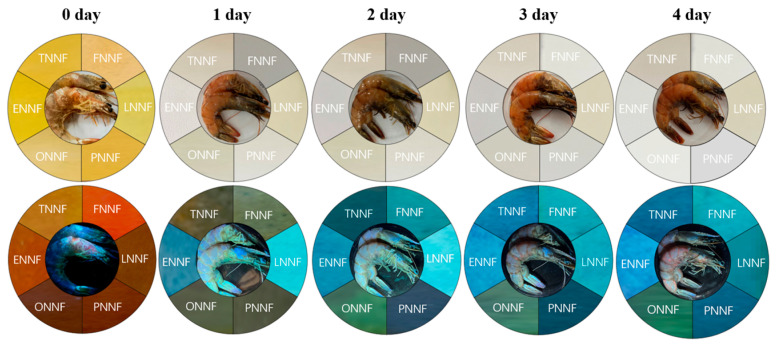
Monitoring fresh shrimp spoilage at 28 °C using BFH-loaded filter paper with colorimetric (**up**) and fluorescent (**bottom**) dual modes.

In protein-rich food storage, protein and amine degradation produces volatile basic nitrogen (TVB-N), a well-established indicator of food freshness that aligns with other spoilage markers, such as sensory evaluations and microbial counts. To monitor spoilage based on amine levels, shrimp was used as a test sample over a one-day storage period. During this period, the fluorescence sensor array exhibited significant color changes (Figure 13) while concurrent measurements of TVB-N values were conducted to assess freshness. These color changes in the fluorescence sensor array enabled clear classification of the shrimp into distinct freshness categories, demonstrating its effectiveness as a spoilage indicator.

**Figure 14:**
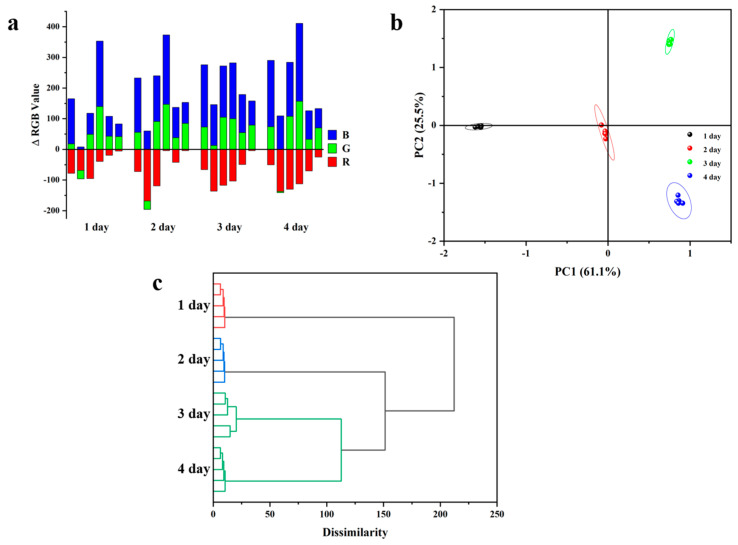
The application of the sensor array to monitor shrimp spoilage. (**a**) RGB color change map of the paper sensor array prepared with BFHs after exposure to shrimp spoilage vapor. (**b**) PCA fraction plot of RGB color change of paper-based sensor array after exposure to shrimp spoilage vapor. (**c**) Hierarchical clustering analysis (HCA) tree diagram of sensor arrays with BFHs exposed to prawn spoilage.

The color changes in the sensing units of the fluorescence sensor array corresponded with the defined freshness categories, displaying a transition from red to green (Figure 14a). For both statistical analyses, variables were represented by the relative change in each color vector (ΔR^2^, ΔG^2^, or ΔB^2^, normalized by the total squared Euclidean distance). A total of 120 parameters (6 dyes × 4 freshness categories (different days) × 5 repetitions) were used for statistical evaluation, with each measurement repeated five times to ensure accuracy.

Figure 14b presents the principal component analysis (PCA) results for sampling days, using data from all dyes across five replicates. The analysis clearly differentiates between sampling days, highlighting the sensor array’s ability to distinguish freshness levels over time. The results of our hierarchical cluster analysis (HCA), which employs both agglomerative and divisive methods to identify clusters within datasets, further support these findings. All 20 cases (4 days × 5 replicates) were correctly assigned to their respective groups without misclassification, demonstrating the array’s strong selectivity in categorizing different sampling days. Additionally, the HCA analysis suggests the potential for expanded application, particularly by developing a database of difference maps for various sampling days, as shown in Figure 14c. This database would enhance the system’s capacity for accurate, time-based freshness assessment.

## 4. Conclusions

In conclusion, a BF_3_-activated fluorescent probe, HL, with sensitive detection and a rapid response time (<2 min), has been successfully synthesized. The probe’s fluorescence activation can be directly observed with the naked eye under sunlight or ultraviolet light. The probe HLs demonstrated superior selectivity for BF_3_ even when other metal ions were present, accompanied by obvious fluorescence enhancement. The probe HLs had a low detection limit of 0.098~0.89 μM for BF_3_. Ultimately, a BF_3_ “OFF-ON-OFF”-type fluorescent probe with industrial application value has been achieved. Additionally, boron difluoride (BF_2_) hydrazone complexes (BFHs) have exhibited versatile response modes due to their multi-color and multi-channel detection capabilities, effectively distinguishing various biogenic amines (BAs) in solution and volatile organic amines (VOAs) in vapor. Sensor arrays based on BFHs have shown promise as reusable and responsive detection platforms. Moreover, chromogenic array platforms have enabled simplified, on-site monitoring of shrimp spoilage, allowing for both visual observation and quantitative analysis. These findings are critical for advancing food safety and quality assessment techniques.

## Data Availability

All data generated or analyzed during this study are included in the published article (and its Supplementary Materials files).

## Supplementary Materials

The following supporting information can be downloaded at https://www.mdpi.com/article/10.3390/s24237415/s1.

## References

[1] Gao L., Zheng M., Liu H., Xiao J., Gong T., Liu K., Li J., Liu J. (2023). A Novel HBI-Based Ratiometric Fluorescent Probe for Rapid Detection of Trifluoroborate. RSC Adv..

[2] Lv B., Chen L., Wang Z., Zheng Y., Cui Z., Wu Y., Li J., Gu W. (2024). A Novel Ratiometric Tanshinone IIA-Based Fluorescent Probe for Sensitive and Reversible Detection of BF3 in Solution and Gaseous Phase. Tetrahedron.

[3] Seenu P., Iyer S.K. (2024). A Phenanthridine-Based Fluorescent Sensor for Selective Ratiometric Detection of BF3 with AIE Properties and Applications in Environmental Monitoring. Dye Pigment..

[4] Liu Y., Zhang J., Wang Y., Liu C., Zhang G., Liu W. (2017). A Rapid and Naked-Eye Visible FRET Ratiometric Fluorescent Chemosensor for Sensitive Detection of Toxic BF3. Sens. Actuators B.

[5] Ahmad W., Mohammed G.I., Al-Eryani D.A., Saigl Z.M., Alyoubi A.O., Alwael H., Bashammakh A.S., O’Sullivan C.K., El-Shahawi M.S. (2020). Biogenic Amines Formation Mechanism and Determination Strategies: Future Challenges and Limitations. Crit. Rev. Anal. Chem..

[6] Jarangdet T., Pratumyot K., Srikittiwanna K., Dungchai W., Mingvanish W., Techakriengkrai I., Sukwattanasinitt M., Niamnont N. (2018). A Fluorometric Paper-Based Sensor Array for the Discrimination of Volatile Organic Compounds (VOCs) with Novel Salicylidene Derivatives. Dye Pigment..

[7] Erdag D., Merhan O., Yildiz B., Proestos C. (2019). Biochemical and Pharmacological Properties of Biogenic Amines. Biogenic Amines.

[8] Heerthana V.R., Preetha R. (2019). Biosensors: A Potential Tool for Quality Assurance and Food Safety Pertaining to Biogenic Amines/Volatile Amines Formation in Aquaculture Systems/Products. Rev. Aquac..

[9] Bekhit A.E.-D.A., Holman B.W.B., Giteru S.G., Hopkins D.L. (2021). Total Volatile Basic Nitrogen (TVB-N) and Its Role in Meat Spoilage: A Review. Trends Food Sci. Technol..

[10] Tang Z., Yang J., Yu J., Cui B. (2010). A Colorimetric Sensor for Qualitative Discrimination and Quantitative Detection of Volatile Amines. Sensors.

[11] Malik R., Tomer V.K., Joshi N. (2018). Au-TiO_2_-Loaded Cubic g-C3N4 Nanohybrids for Photocatalytic and Volatile Organic Amine Sensing Applications. ACS Appl. Mater. Interfaces.

[12] Mohan B., Sarkar D., Raja Lakshmi P., Umadevi D., Shanmugaraju S. (2023). N-Aryl-4-Amino-1,8-Naphthalimide Tröger’s Bases-Based Internal Charge Transfer (ICT) Fluorescence ‘Turn-on’ Chemosensors for Volatile Organic Amines. J. Photochem. Photobiol. A.

[13] Eaidkong T., Mungkarndee R., Phollookin C., Tumcharern G., Sukwattanasinitt M., Wacharasindhu S. (2012). Polydiacetylene Paper-Based Colorimetric Sensor Array for Vapor Phase Detection and Identification of Volatile Organic Compounds. J. Mater. Chem..

[14] Du J., He S., Zhao R., Chen S., Guo T., Wang H. (2017). Facile Self-Assembly of SnO_2_ Nanospheres for Volatile Amine Gas Sensing. Mater. Lett..

[15] Lin C., Wang J., Yang K., Liu J., Ma D.-L., Leung C.-H., Wang W. (2022). Development of a NIR Iridium(III) Complex for Self-Calibrated and Luminogenic Detection of Boron Trifluoride. Spectrochim. Acta A. Mol. Biomol. Spectrosc..

[16] Rice S.L., Eitenmiller R.R., Koehler P.E. (1976). Biologically Active Amines in Food: A Review. J. Milk Food Technol..

[17] Li Z., Hou S., Zhang H., Song Q., Wang S., Guo H. (2023). Recent Advances in Fluorescent and Colorimetric Sensing for Volatile Organic Amines and Biogenic Amines in Food. Adv. Agrochem..

[18] Li M. (2024). Deep Learning-Assisted Flavonoid-Based Fluorescent Sensor Array for the Nondestructive Detection of Meat Freshness. Food Chem..

[19] Hu J., Liu R., Zhai S., Wu Y., Zhang H., Cheng H., Zhu H. (2017). AIE-Active Molecule-Based Self-Assembled Nano-Fibrous Films for Sensitive Detection of Volatile Organic Amines. J. Mater. Chem. C.

[20] Aresta A., Cotugno P., De Vietro N., Longo C., Mercurio M., Ferriol P., Zambonin C., Nonnis Marzano C. (2021). Volatile Organic Compounds, Indole, and Biogenic Amines Assessment in Two Mediterranean Irciniidae (Porifera, Demospongiae). Mar. Drugs.

[21] Liu H., Zhang Y., Huang L., Wang M. (2022). A Colorimetric Gas-Sensitive Array Sensor Using Filter Paper for the Analysis of Fish Freshness. Food Chem..

[22] Xu X., Wang X., Ding Y., Zhou X., Ding Y. (2024). Integration of Lanthanide MOFs/Methylcellulose-Based Fluorescent Sensor Arrays and Deep Learning for Fish Freshness Monitoring. Int. J. Biol. Macromol..

[23] Li Y., Ma Y., Huang T., Huang J., Shen K., Li G., Huang M. (2024). An AIE and ESIPT Based Ratiometric Fluorescent Hydrogel Sensor for Detecting Biogenic Amines and Chicken Breast Freshness. Microchem. J..

[24] Salinas Y., Ros-Lis J.V., Vivancos J.-L., Martínez-Máñez R., Marcos M.D., Aucejo S., Herranz N., Lorente I. (2012). Monitoring of Chicken Meat Freshness by Means of a Colorimetric Sensor Array. Analyst.

[25] Wang D. (2024). Intelligent Vegetable Freshness Monitoring System Developed by Integrating Eco-Friendly Fluorescent Sensor Arrays with Deep Convolutional Neural Networks. Chem. Eng. J..

[26] Romero-González R., Alarcón-Flores M.I., Vidal J.L.M., Frenich A.G. (2012). Simultaneous Determination of Four Biogenic and Three Volatile Amines in Anchovy by Ultra-High-Performance Liquid Chromatography Coupled to Tandem Mass Spectrometry. J. Agric. Food Chem..

[27] Wang L., Ran X., Tang H., Cao D. (2021). Recent Advances on Reaction-Based Amine Fluorescent Probes. Dye Pigment..

[28] Zhai L., Zhang F., Sun J., Liu M., Sun M., Lu R. (2017). New Non-Traditional Organogelator of β-Diketone-Boron Difluoride Complexes with Terminal Tetraphenylethene: Self-Assembling and Fluorescent Sensory Properties towards Amines. Dye Pigment..

[29] Shen A., Hao X., Zhang L., Du M., Li M., Yuan J., Du X., Ma S., Zhao Y., Hou L. (2022). Heptamethine Cyanine Dye-Based Functionalized Recyclable Flexible Cotton Fabric Platform for the Colorimetric and Real-Time Ultrasensitive Detection of Gaseous BF3. Dye Pigment..

[30] Zhang P., Wu T., Cao H., Zhang J., James T.D., Sun X. (2023). Fluorometric Detection of Volatile Amines Using an Indanonalkene Platform. Org. Chem. Front..

[31] Xiao M. (2024). Functionalized Carbon Quantum Dots Fluorescent Sensor Array Assisted by a Machine Learning Algorithm for Rapid Foodborne Pathogens Identification. Microchem. J..

[32] Zhao J.-H., Xu W.-X., Li B., Xu W., Zhang W.-K., Yuan M.-S., Li H.-Z., He Q.-G., Ma X., Cheng J.-G. (2024). Portable Fluorogenic Probe for Monitoring of Volatile Amine Vapour and Food Spoilage. Chin. Chem. Lett..

[33] Krishnan U., Manickam S., Iyer S.K. (2023). BF3 Detection by Pyrazolo-Pyridine Based Fluorescent Probe and Applications in Bioimaging and Paper Strip Analysis. J. Mol. Liq..

[34] Wang Z., Zhang Y., Song J., Wang Y., Li M., Yang Y., Xu X., Xu H., Wang S. (2020). Two Ultrafast Responsive Isolongifolanone Based Fluorescent Probes for Reversible and Sensitive Visualization of Toxic BF3 in Solution and in Gas Phase. Sens. Actuators B Chem..

[35] Duan L., Wang S., Zhao H., Wu T., Li D., Guo L., Liang X., Sun Y., Guo K., Li J. (2021). Sectional Intramolecular Charge Transfer Manipulating in a D-A-D’ Coumarin Derivative for Recessive Rewritable Paper. Dye Pigment..

[36] Choi M.G., Lee S.H., Jung Y., Hong J.M., Chang S.-K. (2017). Fluorescence Signaling of BF3 Species by Transformation of an ESIPT Dye to Its Difluoroboron Adduct. Sens. Actuators B Chem..

[37] Krishnan U., Manickam S., Kulathu Iyer S. (2023). Selective Detection of BF3 in Living Cells and Environmental Water Samples Using Schiff-Base Fluorescent Probe. J. Photochem. Photobiol. A Chem..

[38] Wang Y., Yang Y., Qiu F., Feng Y., Song X., Zhang G., Liu W. (2018). A Reversible and Colorimetric Fluorescence Probe for Highly Sensitive Detection of Toxic BF3 in Air. Sens. Actuators B Chem..

