# Supplementary material for: The Fluorescent Sensing of BF3 and Amines: A Dual Approach with Hydrazone Ligands
Source: Sensors (Basel). 2024 Nov 21;24(23):7415. doi: 10.3390/s24237415 (PMC11644717; doi:10.3390/s24237415)
Supplement: Supplementary file 1 [file sensors-24-07415-s001.zip › sensors-3317824-supplementary.pdf]

Supporting information for

The Fluorescent Sensing of BF_3 and Amines: A Dual-Approach with Hydrazone Ligands

Haichao Ye, Liqin Liu, Dagang Shen, Chang Song and Huanhuan Wang*

College of Chemistry and Chemical Engineering, Xinjiang Agricultural University, Urumqi 830002, China.

* Corresponding author: Huanhuan Wang, e-mail: wanghuanhuan@xjau.edu.cn(H.W.)

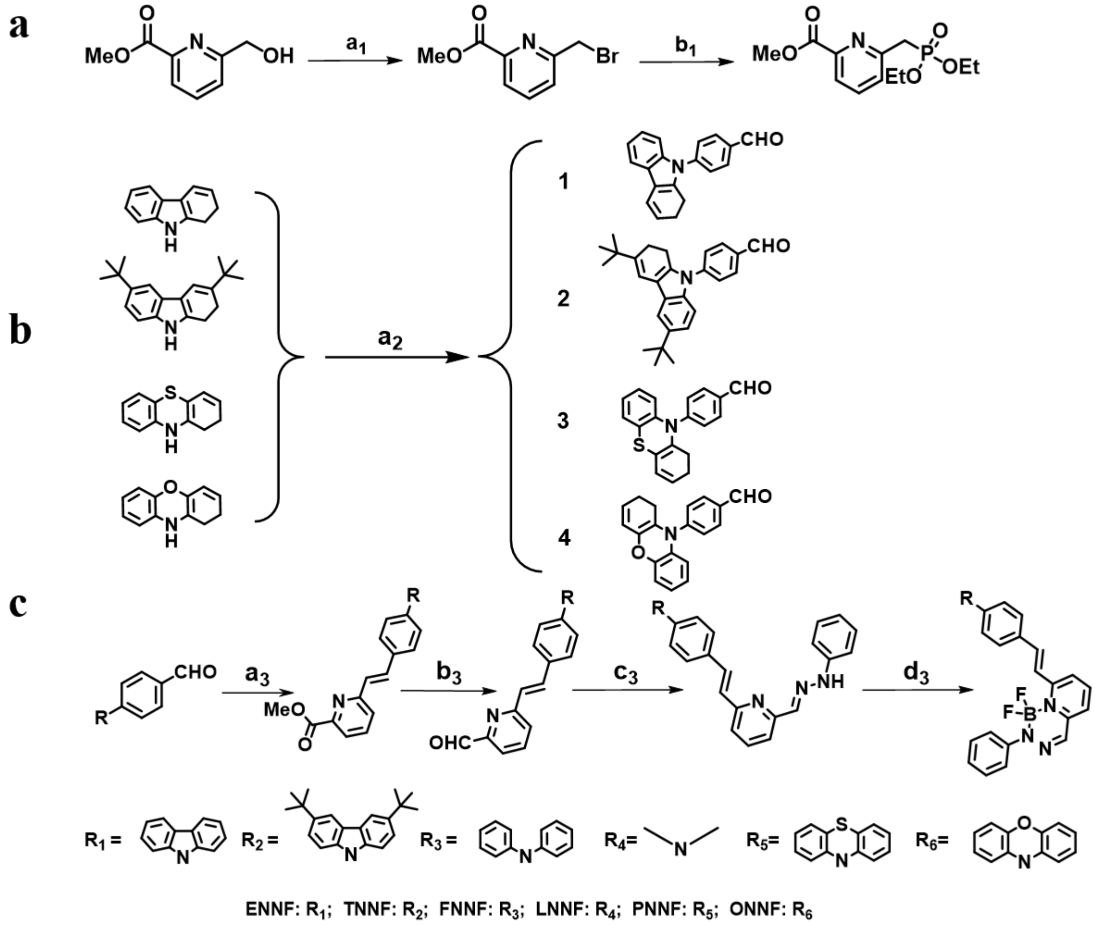

Scheme S1. Molecular synthesis route.

(a)a_1: PBr_3, $CHCl_2$, 0℃.,8h;b_1: Triethyl phosphite,80 ℃,12h;

(b)a_2: 4-Fluorobenzaldehyde, K_2CO_3/K_3PO_4, DMF,120 ℃, 12h;

(c)a_3: Methyl 6-((diethoxyphosphoryl)methyl)picolinate, t-BuOK, THF,-10 ℃, 2h; b_3: DIBAL-H, Toluene, -78 ℃, 2h, N_2; c_3: Phenyl hydrazine, MeOH, Acetic Acid, 65 ℃, 3h; d_4:$BF_3 \cdot OEt_2$, $CHCl_2$, R.T.,30min.

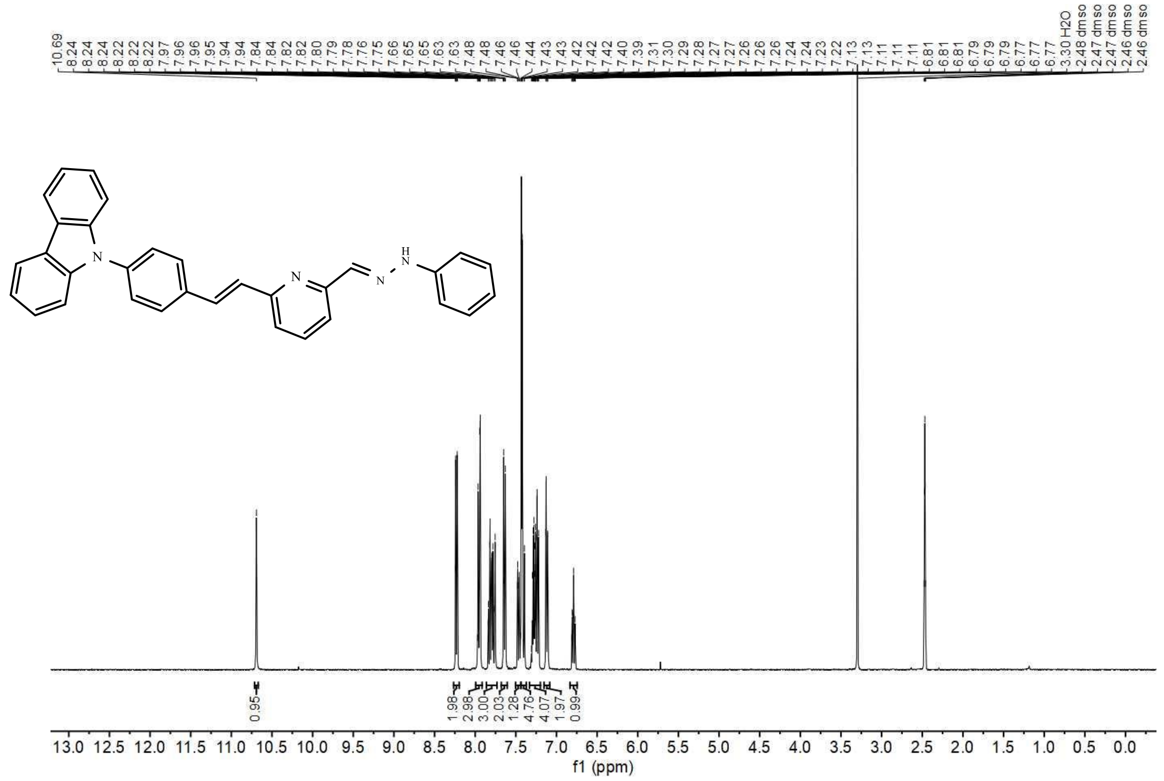

Figure S1. ^{1}H-NMR spectrum of ENN in $(CD_3)_2SO$.

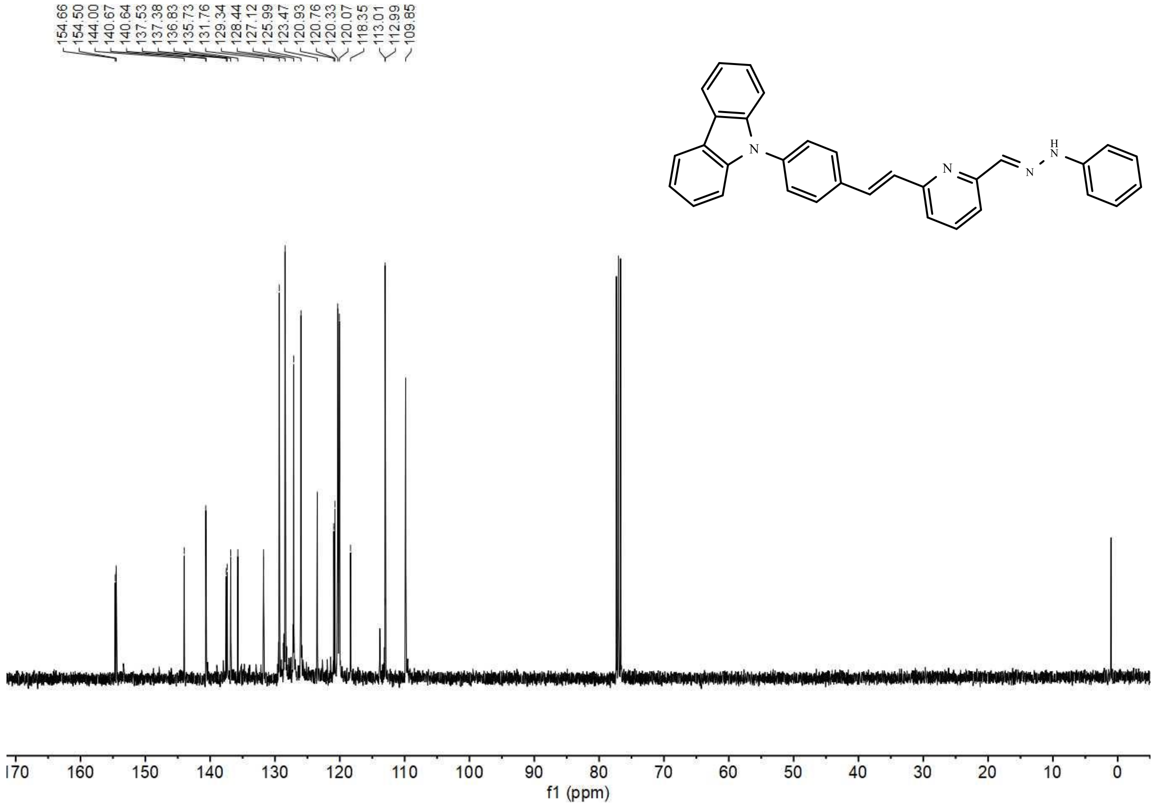

Figure S2. ^{13}C-NMR spectrum of ENN in $CDCl_3$.

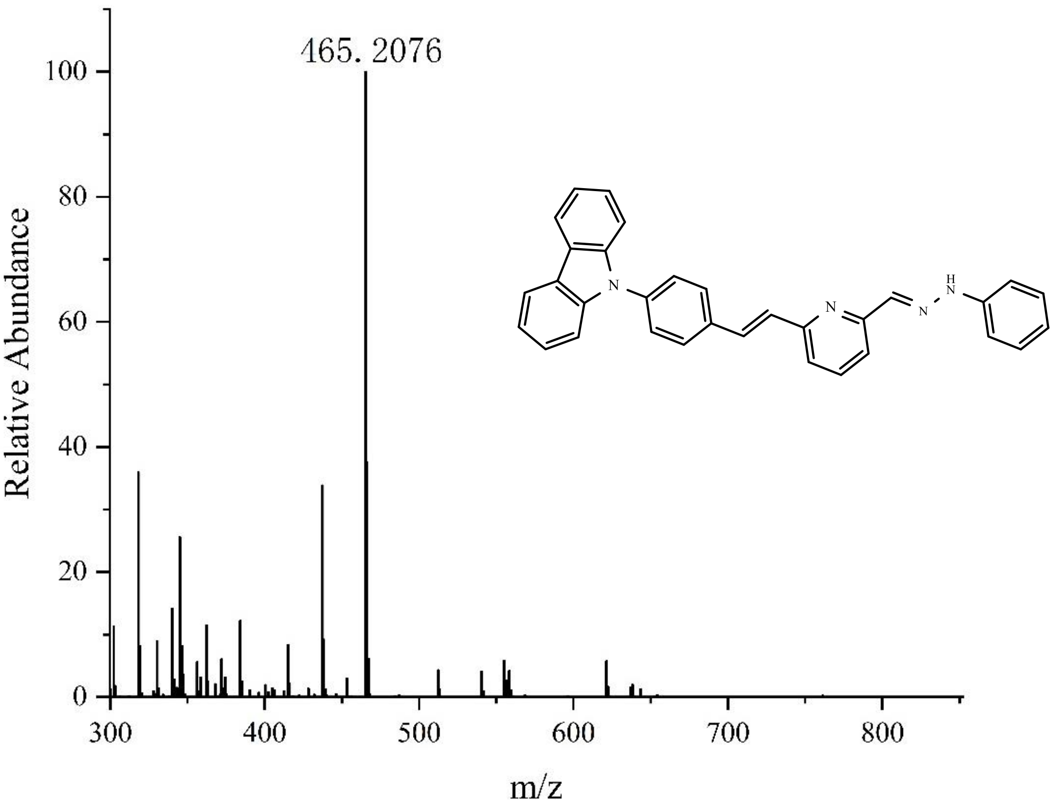

Figure S3. HRMS spectrum of ENN.

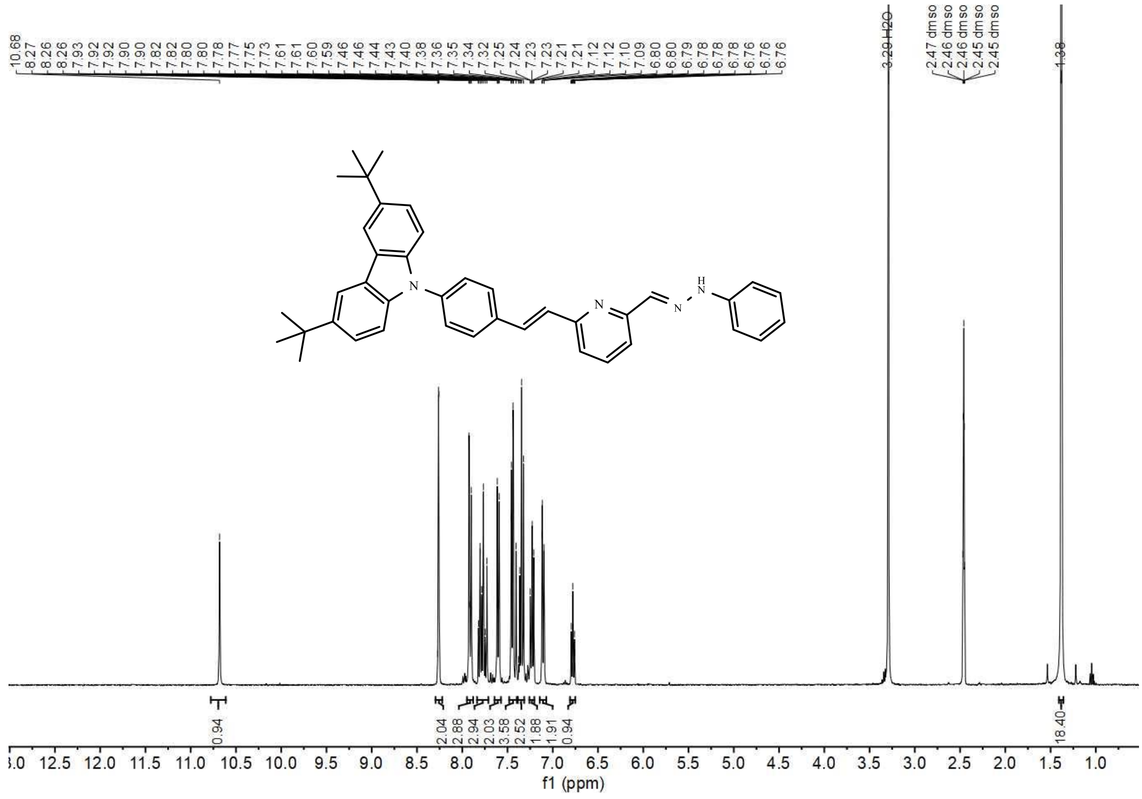

Figure S4. ^{1}H-NMR spectrum of TNN in $(CD_3)_2SO$.

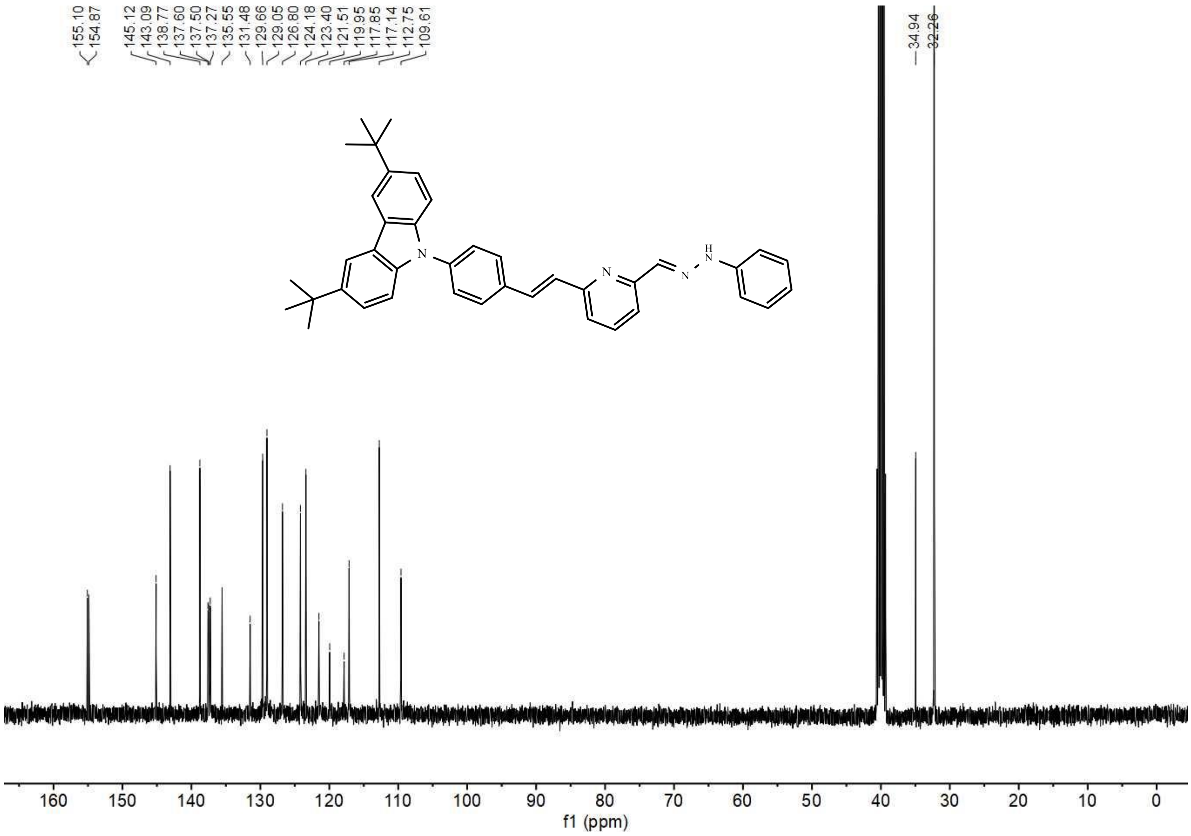

Figure S5. ^{13}C-NMR spectrum of TNN in $(CD_3)_2SO$.

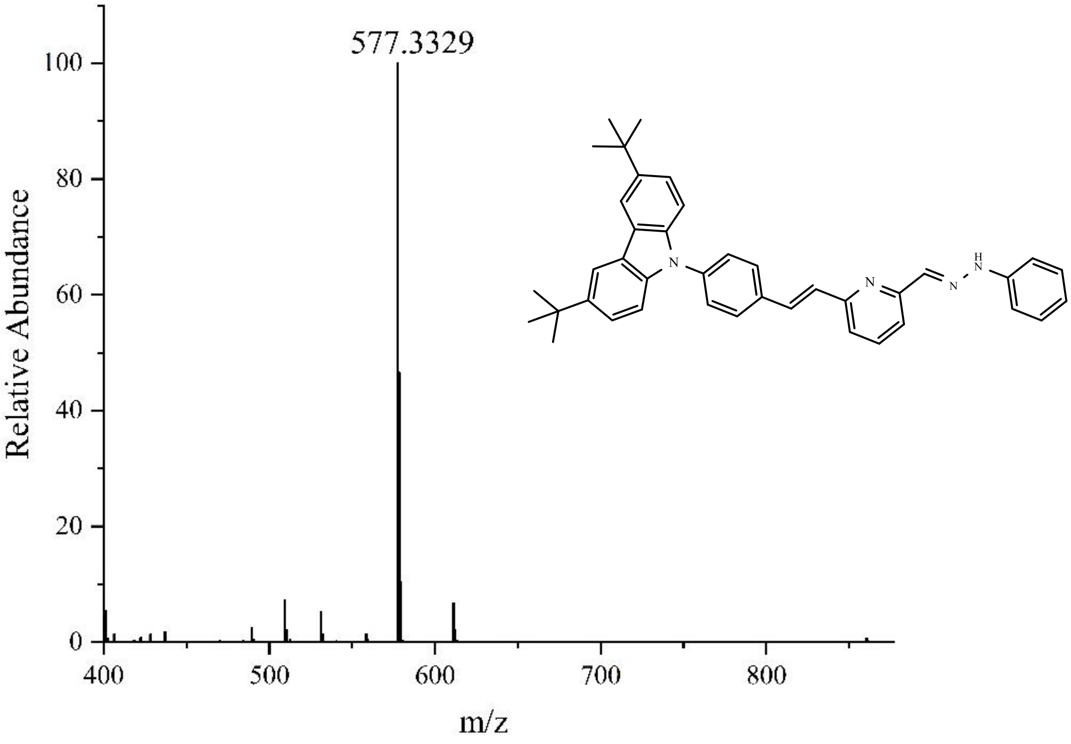

Figure S6. HRMS spectrum of TNN.

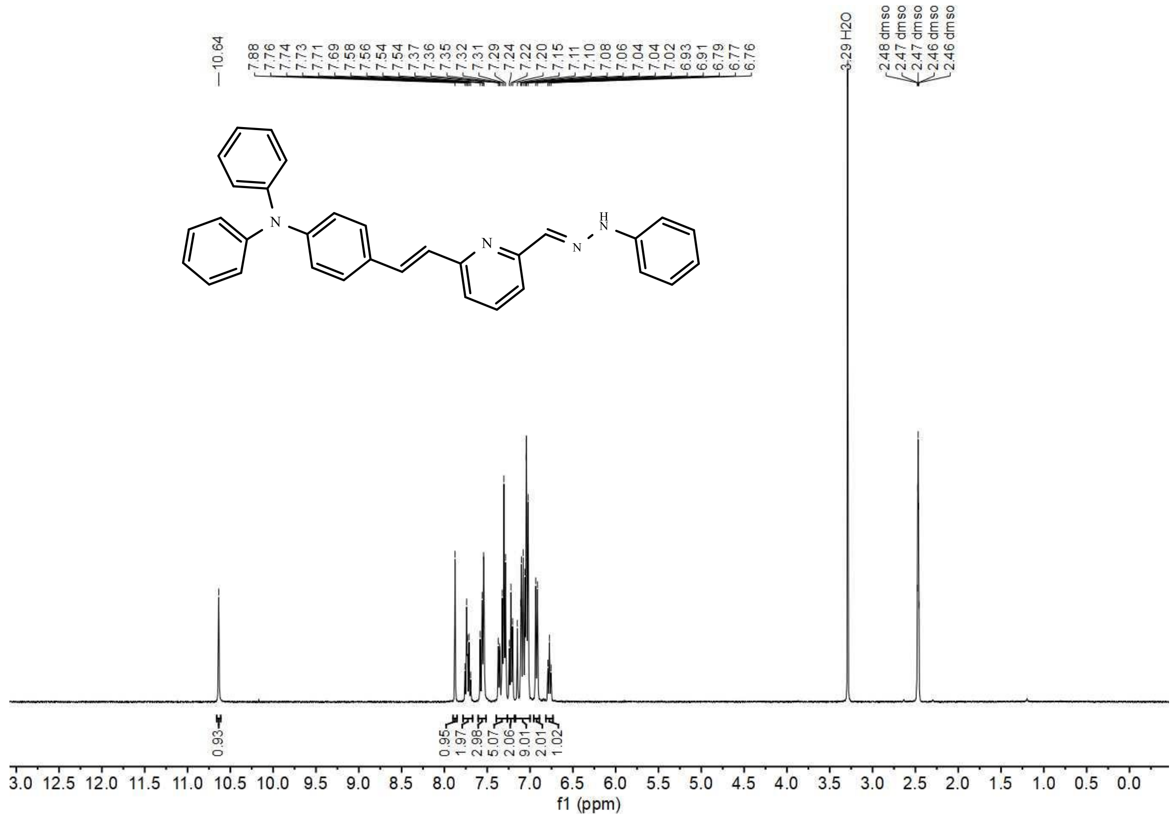

Figure S7. ^{1}H-NMR spectrum of FNN in $(CD_3)_2SO$.

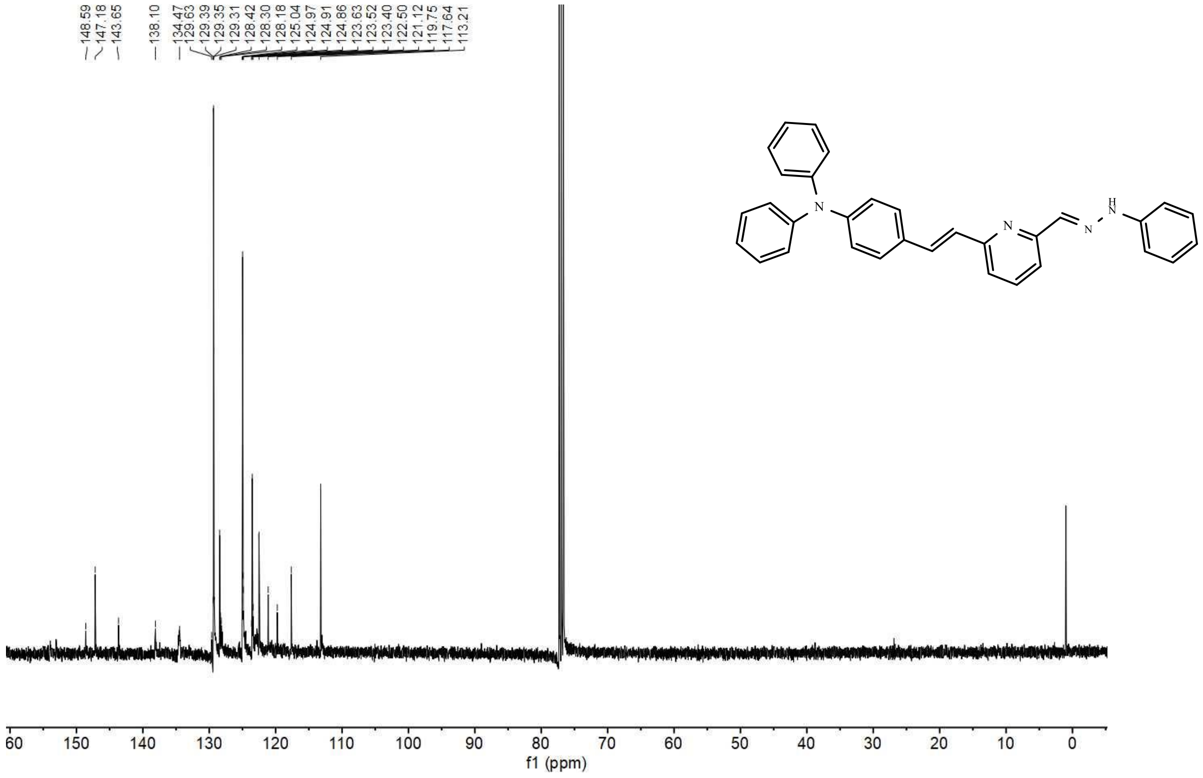

Figure S8. ^{13}C-NMR spectrum of FNN in $CDCl_3$.

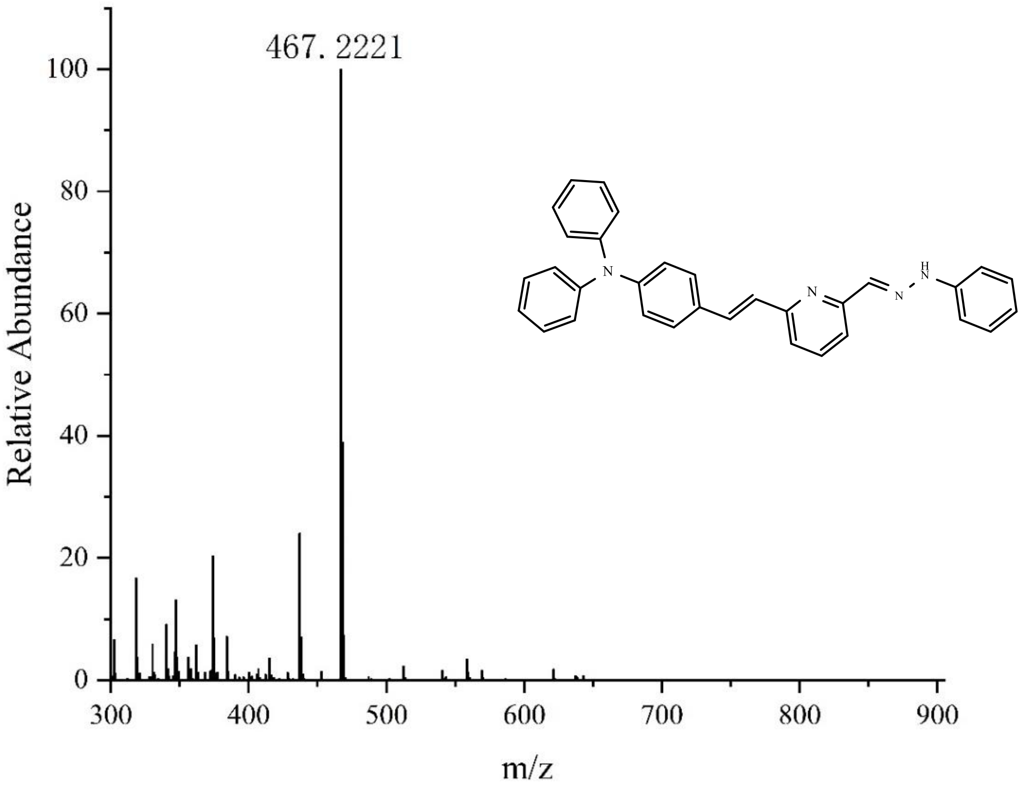

Figure S9. HRMS spectrum of FNN.

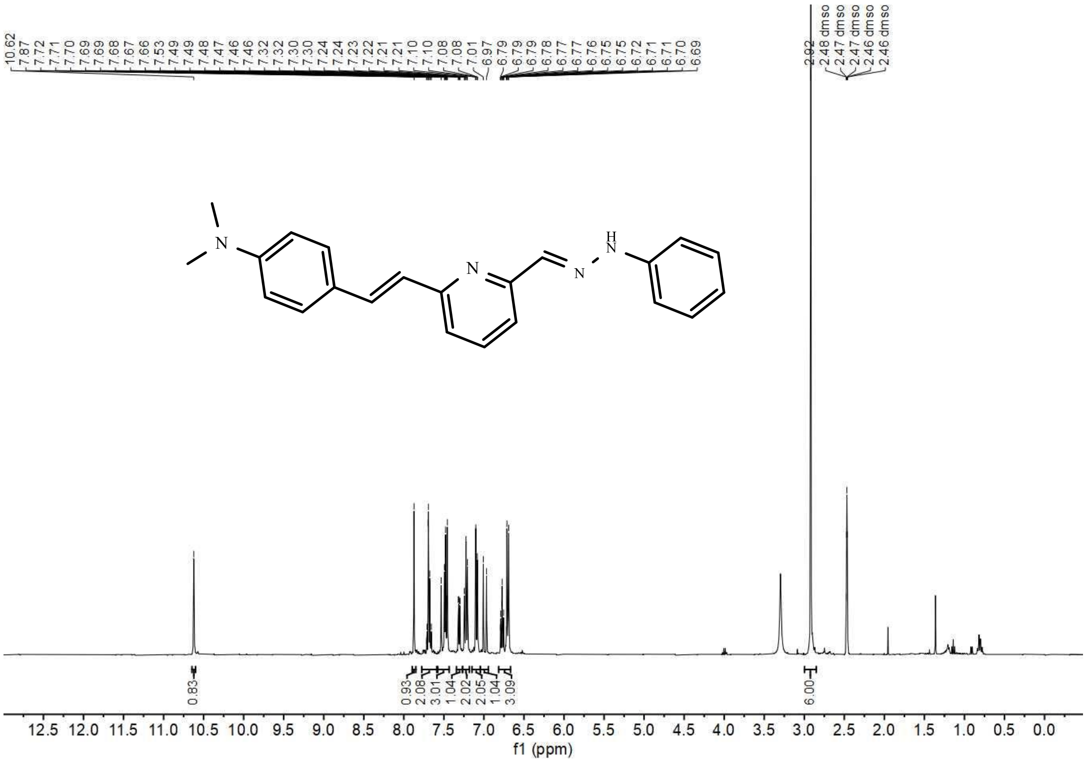

Figure S10. ^{1}H-NMR spectrum of LNN in $(CD_3)_2SO$.

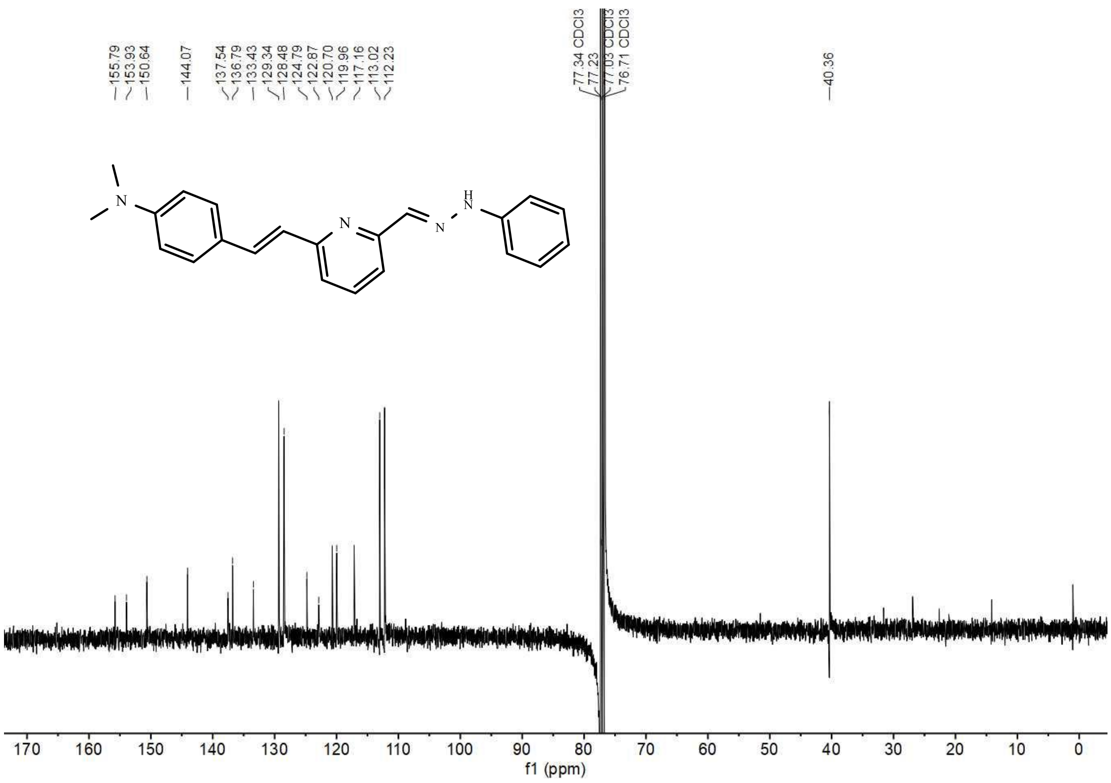

Figure S11. ^{13}C-NMR spectrum of LNN in $CDCl_3$.

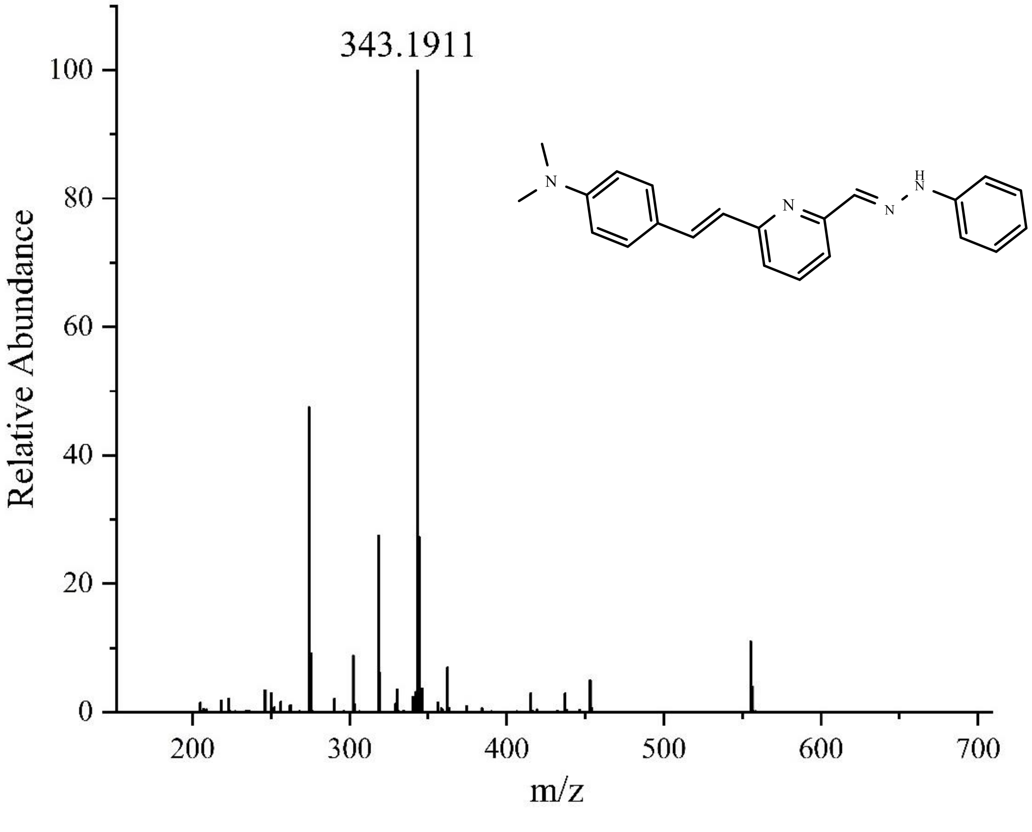

Figure S12. HRMS spectrum of LNN.

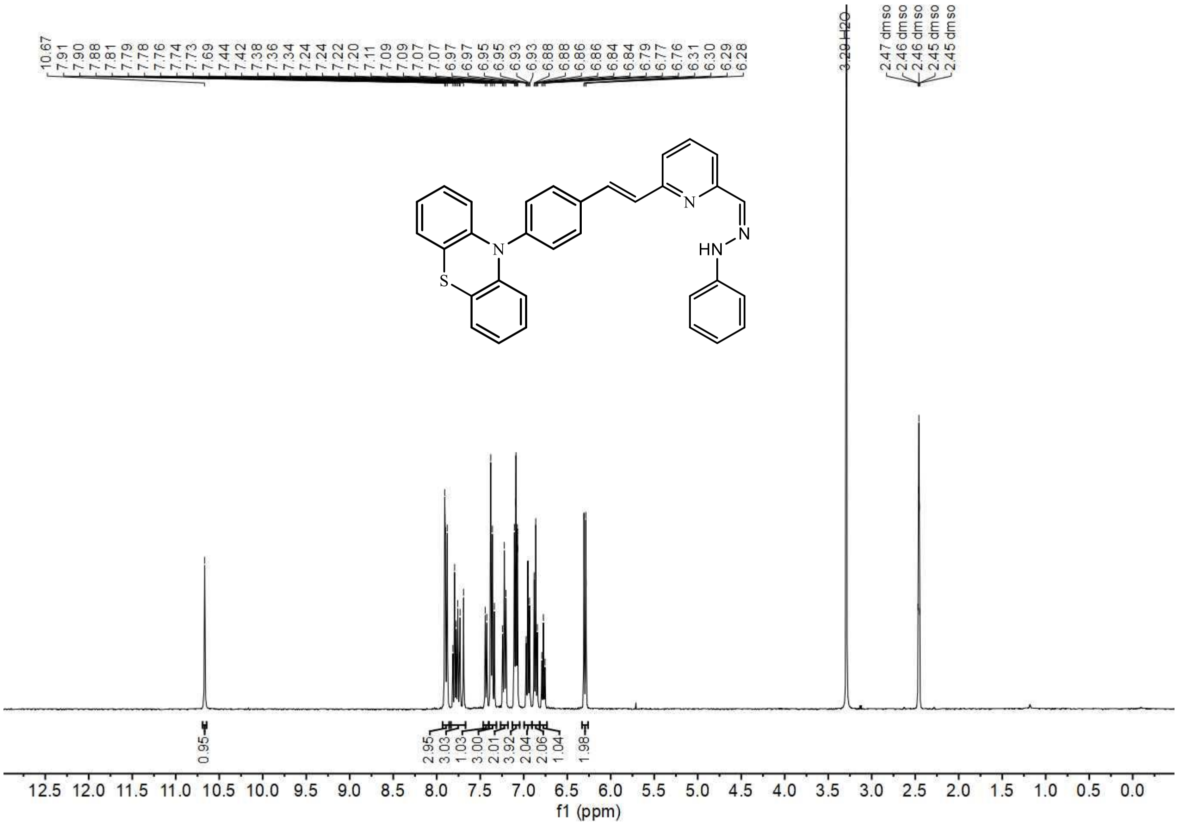

Figure S13. ^{1}H-NMR spectrum of PNN in $(CD_3)_2SO$.

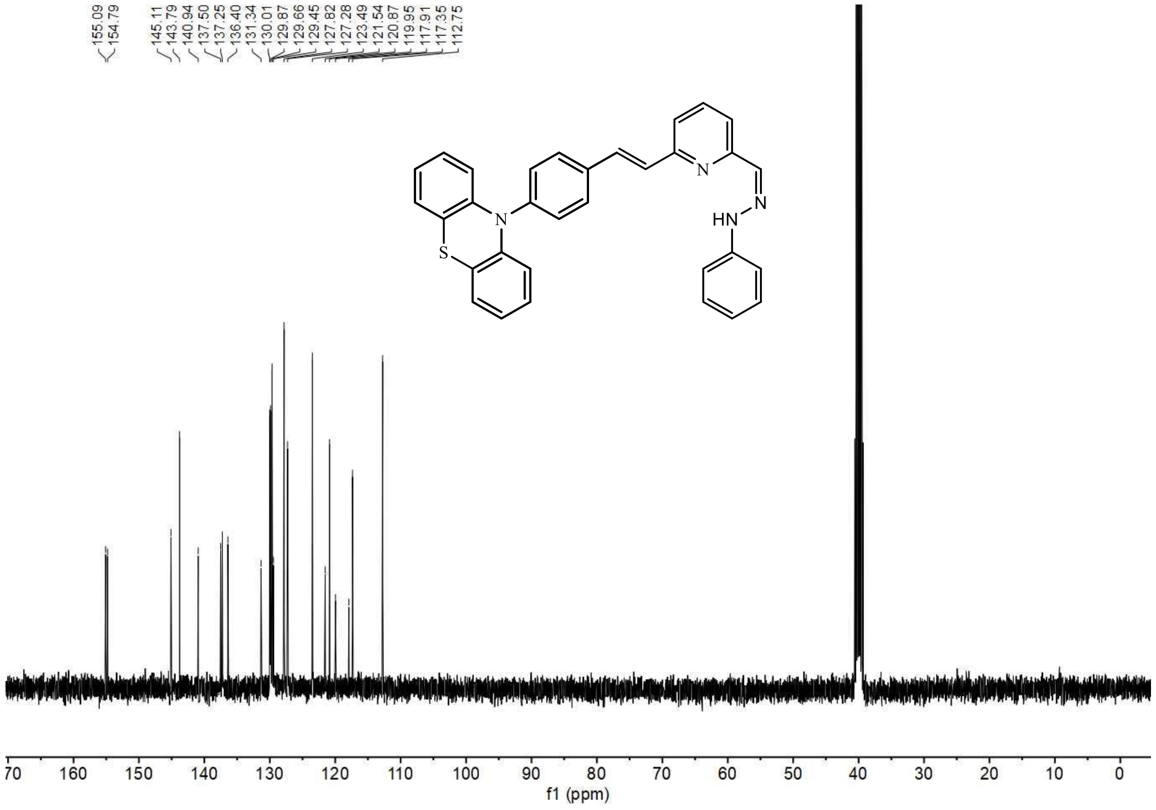

Figure S14. ^{13}C-NMR spectrum of PNN in $(CD_3)_2SO$.

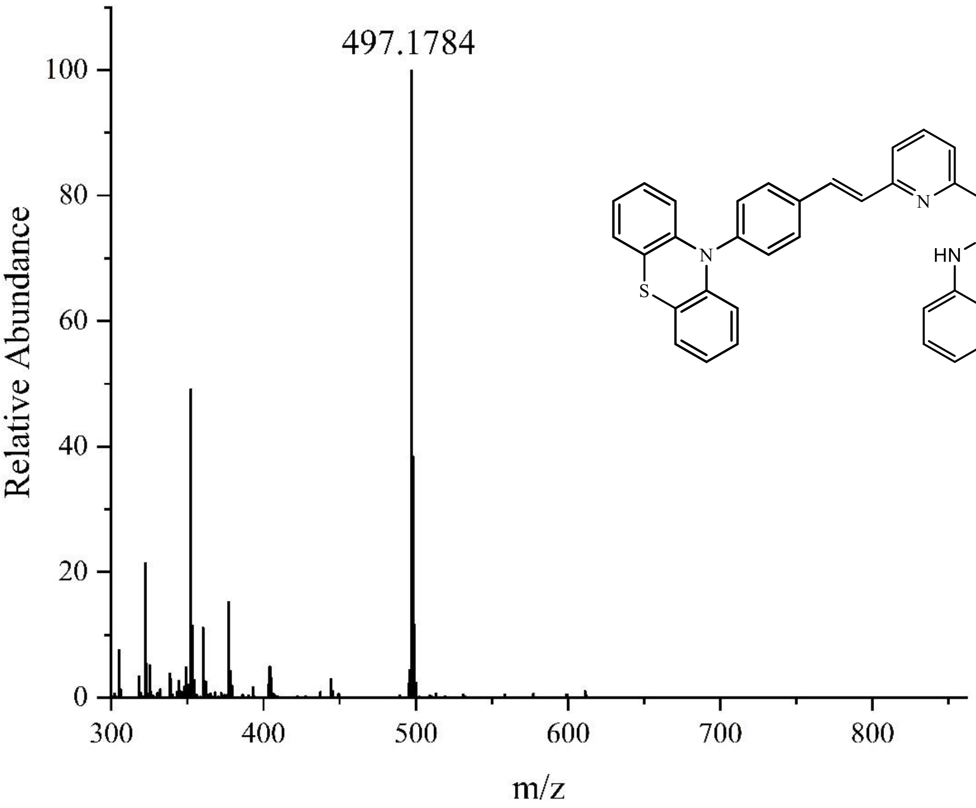

Figure S15. HRMS spectrum of LNN.

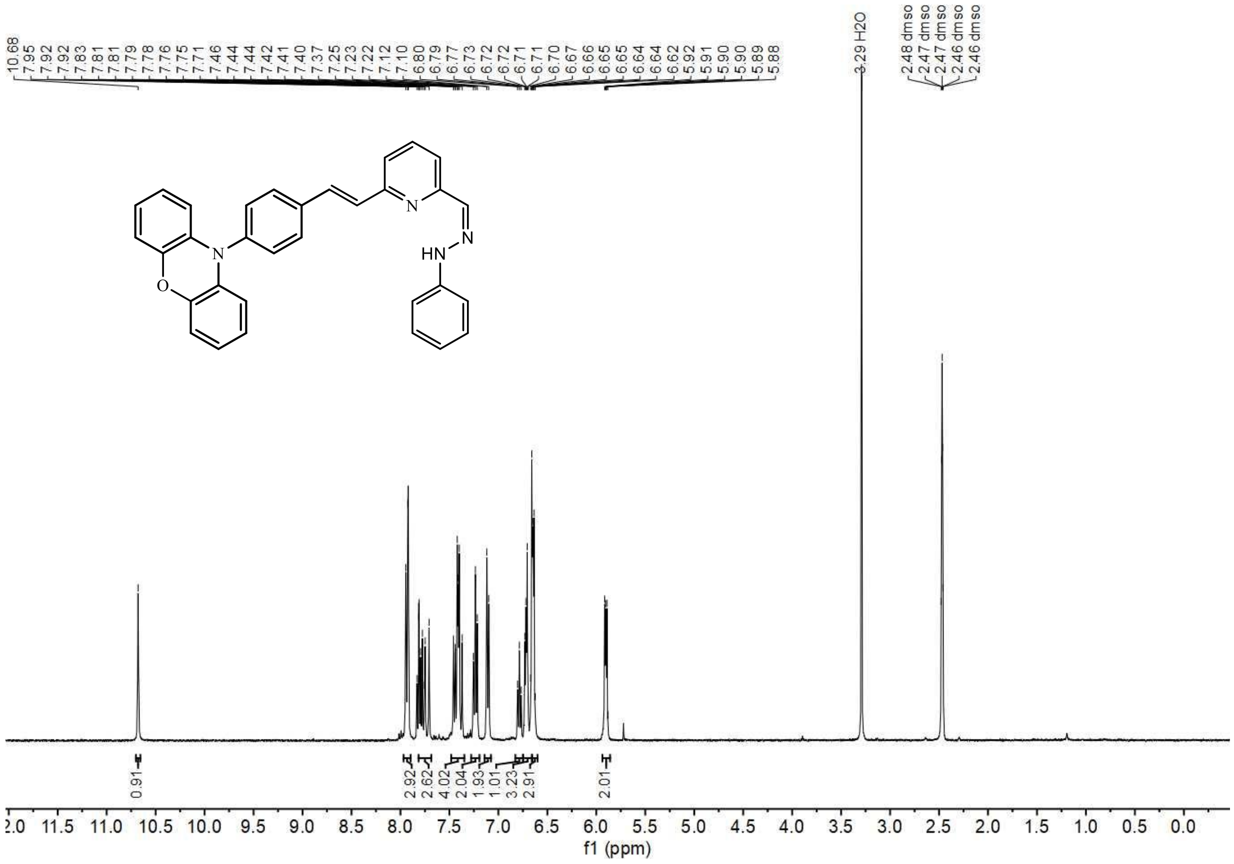

Figure S16. ^{1}H-NMR spectrum of ONN in $(CD_3)_2SO$.

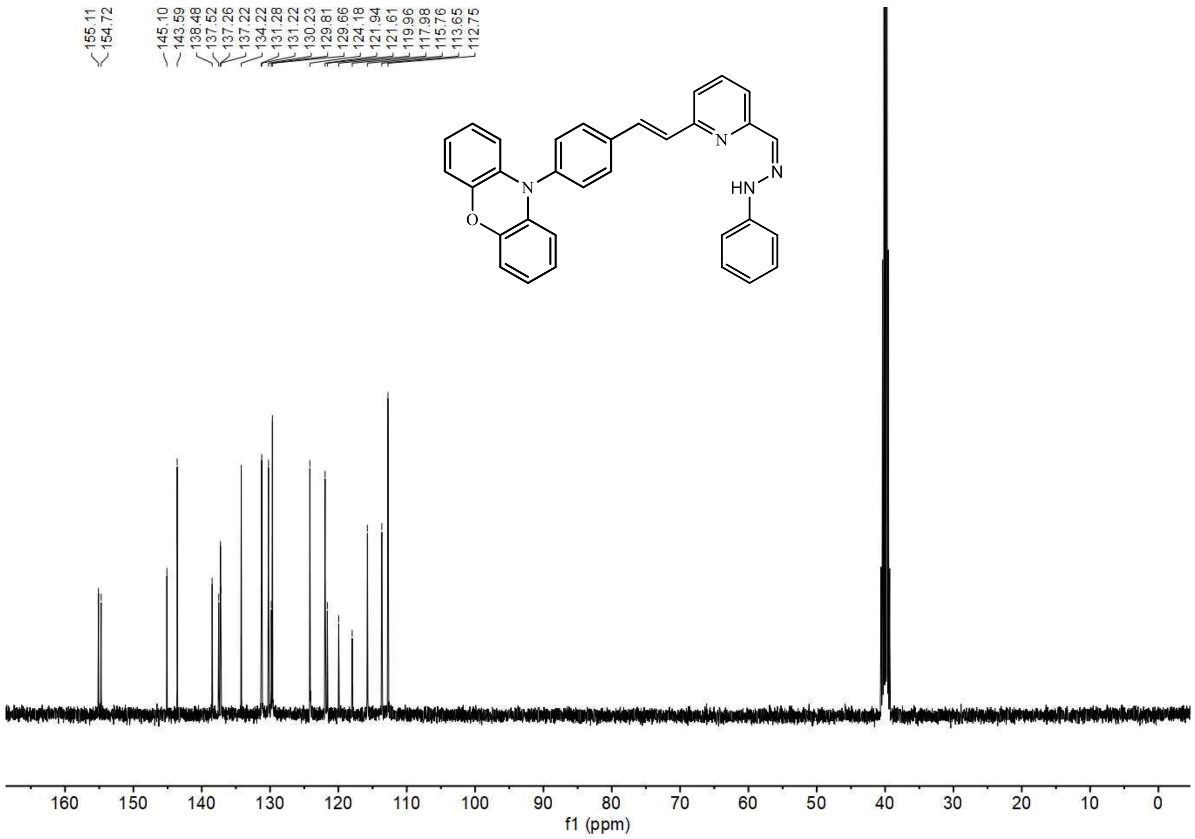

Figure S17. ^{13}C-NMR spectrum of ONN in $(CD_3)_2SO$.

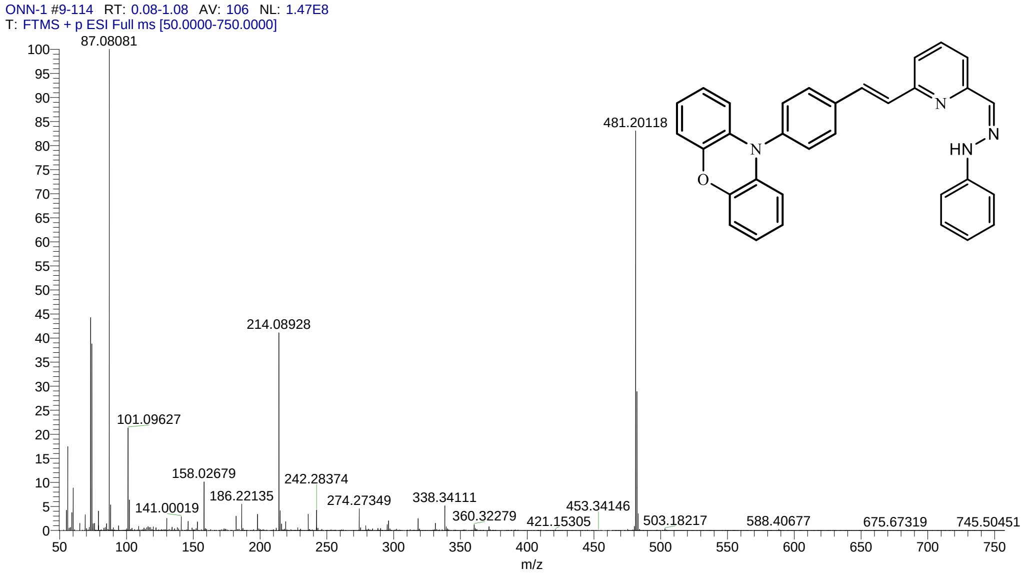

Figure S18. HRMS spectrum of ONN.

The activation of BF_3 signaling in BFHs through the formation of a difluoroboron adduct via N,N coordination exemplifies a commonly employed method for synthesizing other difluoroboron adducts. As demonstrated in Figure S19, the ^{19}F NMR signal of BF_3 shifted from -146.26 ppm to -148.07 ppm, indicating a change in the chemical environment caused by the coordination of hydrazone ligands with BF_3. Moreover, a significant change observed in the ^{1}H NMR spectrum, as compared to the ligands, was the disappearance of the hydroxyl proton resonance around 10.69 ppm, attributable to the electron-deficient boron (Figure S20).

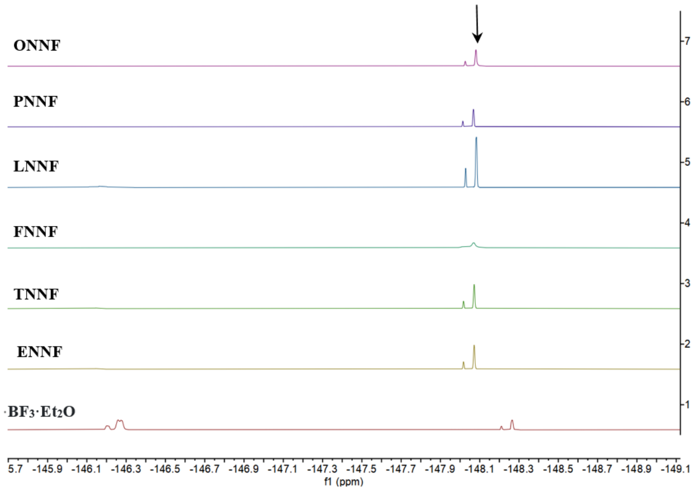

Figure S19. ^{19}F NMR of BF_3 and HLs coordination.

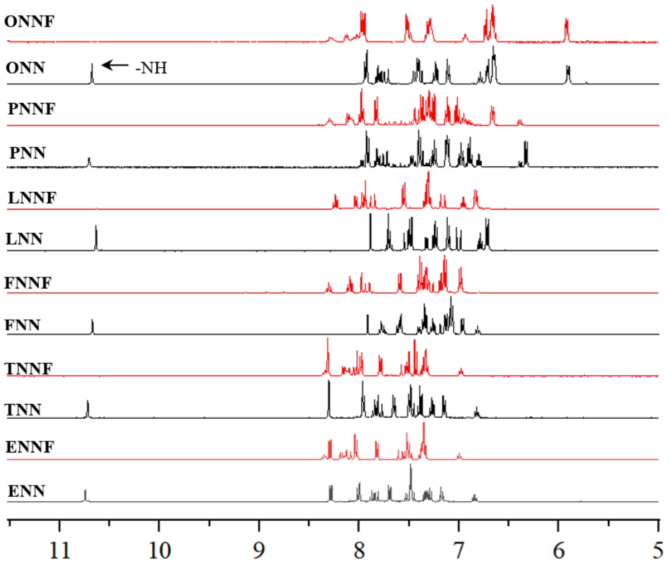

Figure S20. Partial ^{1}H NMR spectra of HLs in the absence and presence of $BF_3{\cdot}Et_2O$. HLs = 5.0 × 10^{-3} M, [$BF_3{\cdot}Et_2O$] = 1.0 × 10^{-2} M in $CDCl_3$.

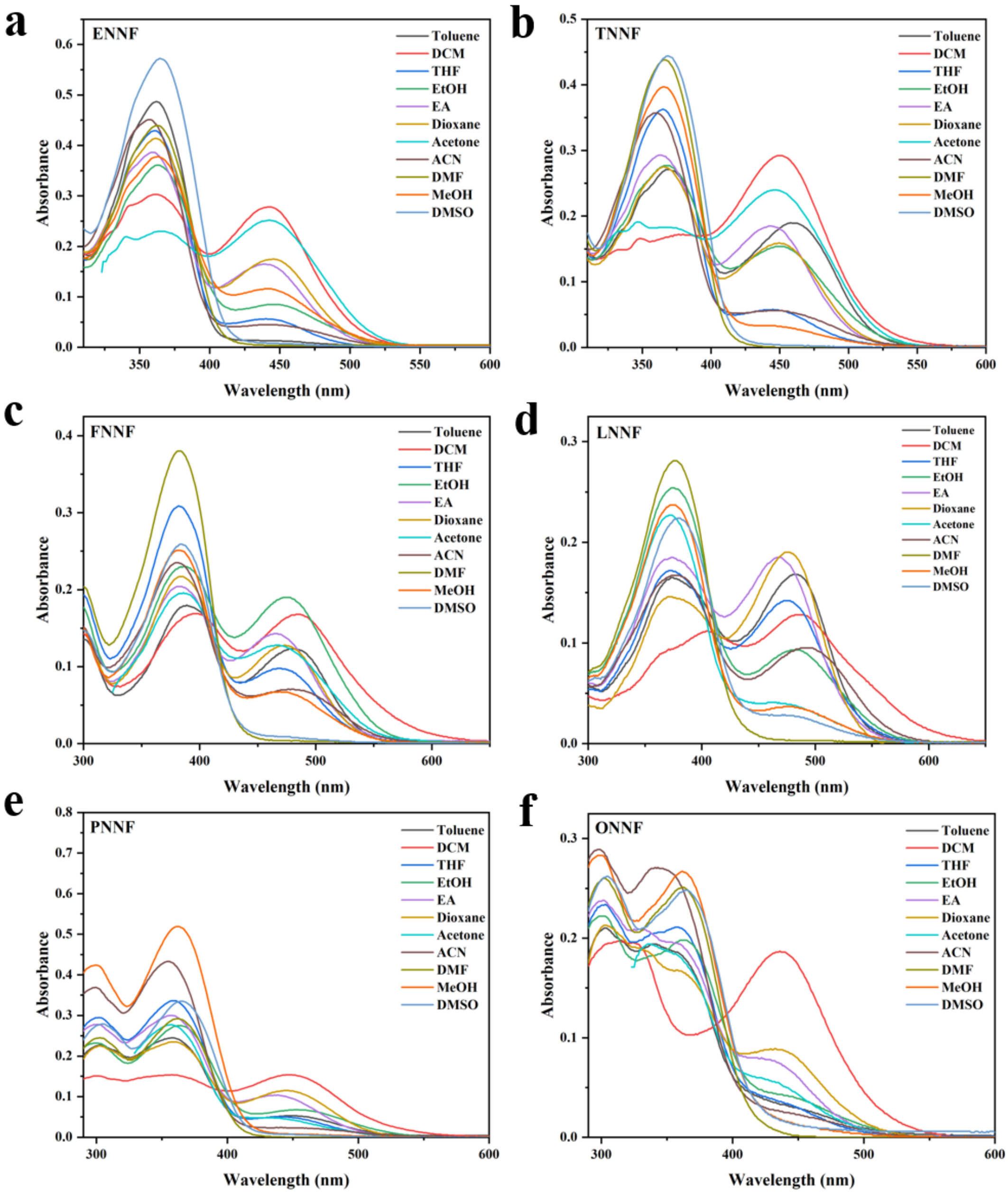

Figure S21. (a–f) UV-Vis spectra of six HLs (5 μM) in the presence of BF_3 (40 μM) in different solvents.

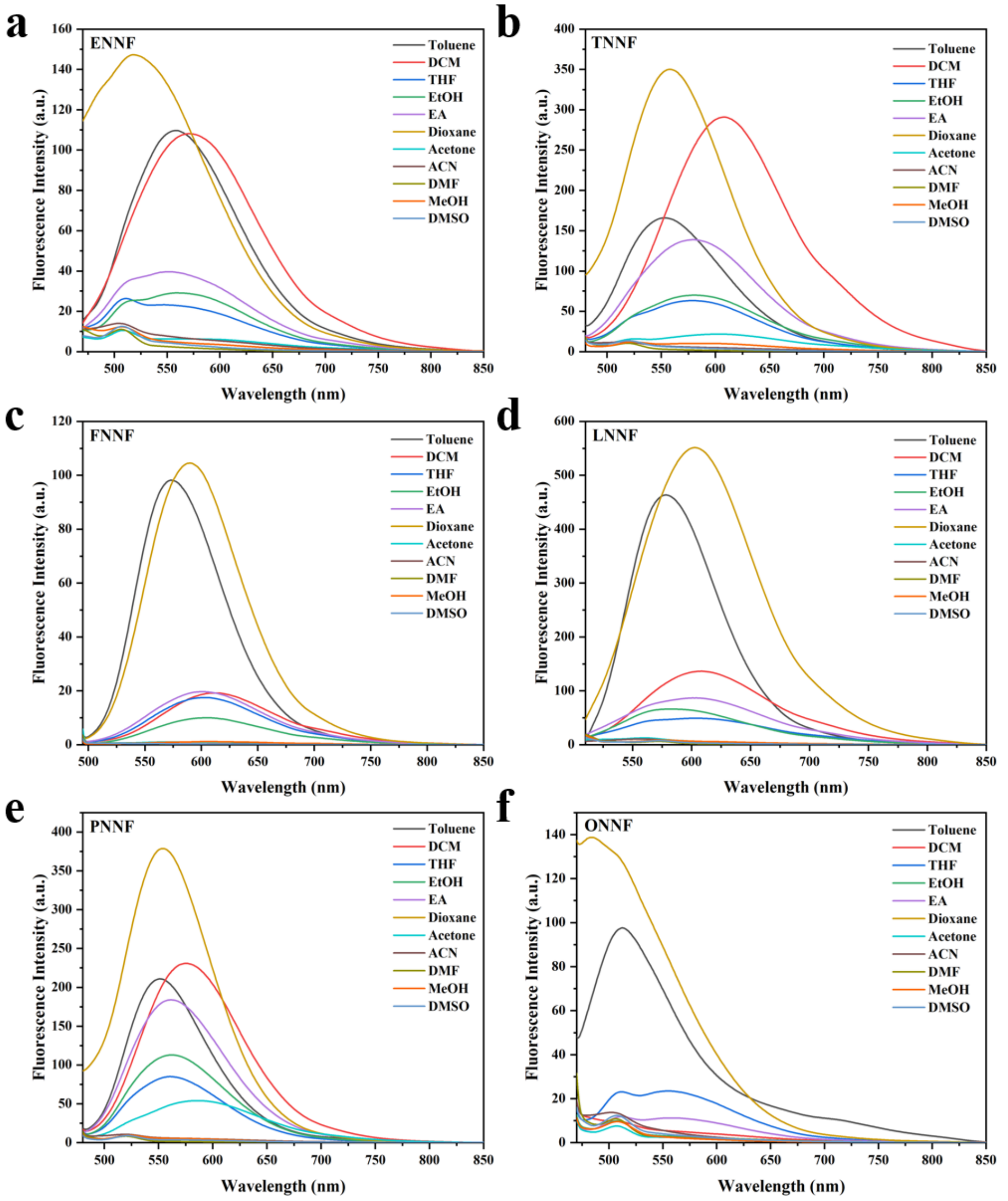

Figure S22. (a–f) Fluorescence intensity of HLs (5 μM) in the presence of BF_3 (40 μM) in different solvents. λ_{ex} = 460 nm.

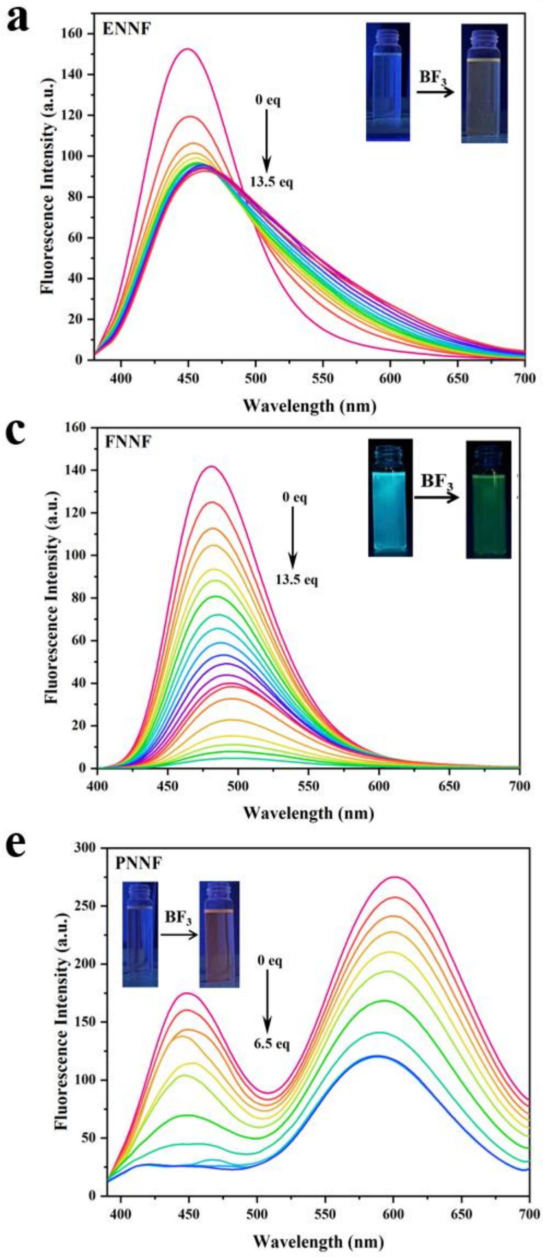

Figure S23. (a–f) Fluorescence spectra of HLs (5 μM) with the addition of BF_3 at different concentrations (0–13.5 eq) in CH_2Cl_2. The excitation wavelength was 365 nm.

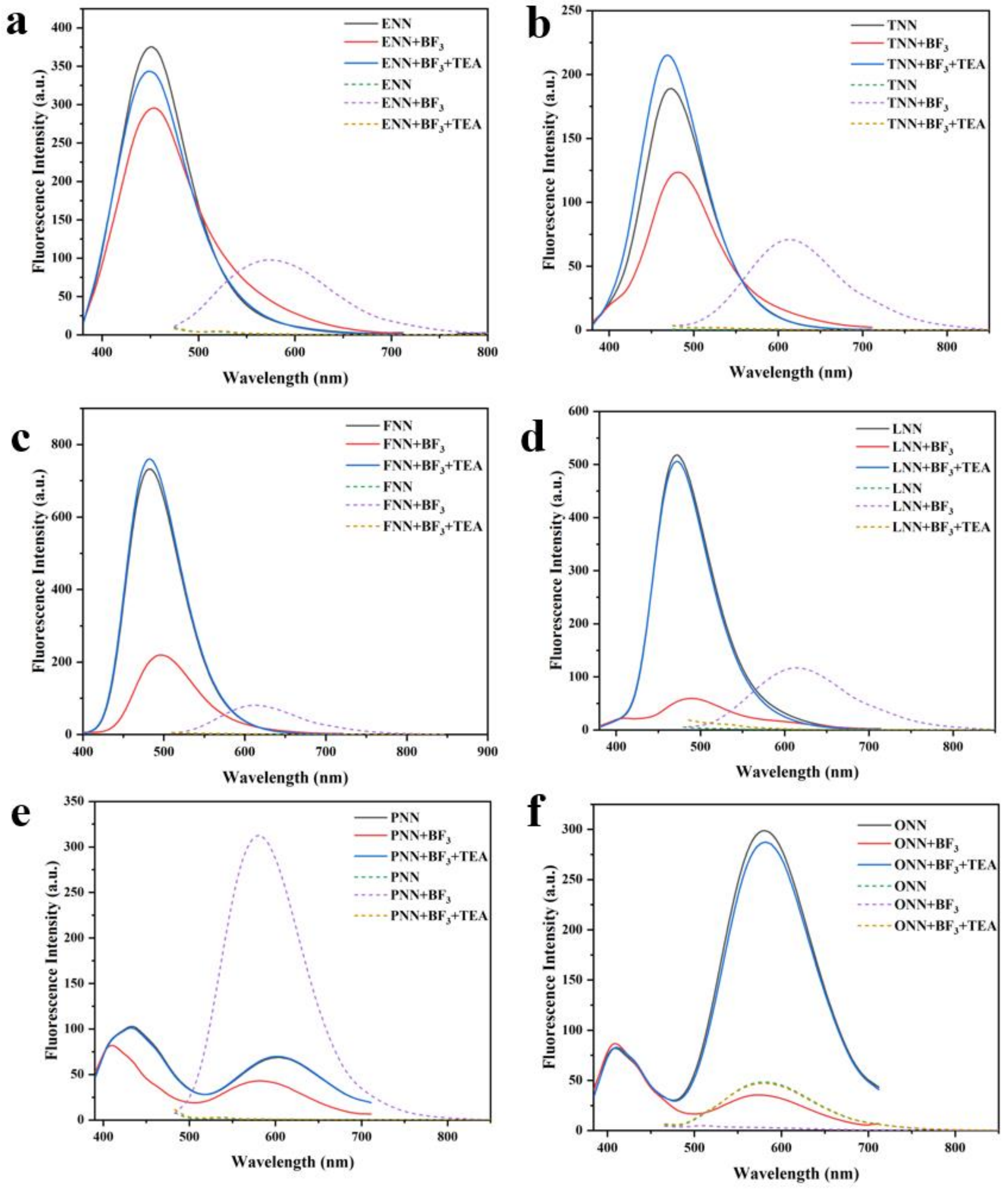

Figure S24. (a–f) Fluorescence spectra of HLs (5 μM) + BF_3 (20 equiv) followed by addition of Et_3N (20 equiv) in CH_2Cl_2 (a-f). The excitation wavelengths of solid line and dotted line were 365 nm and 460nm.

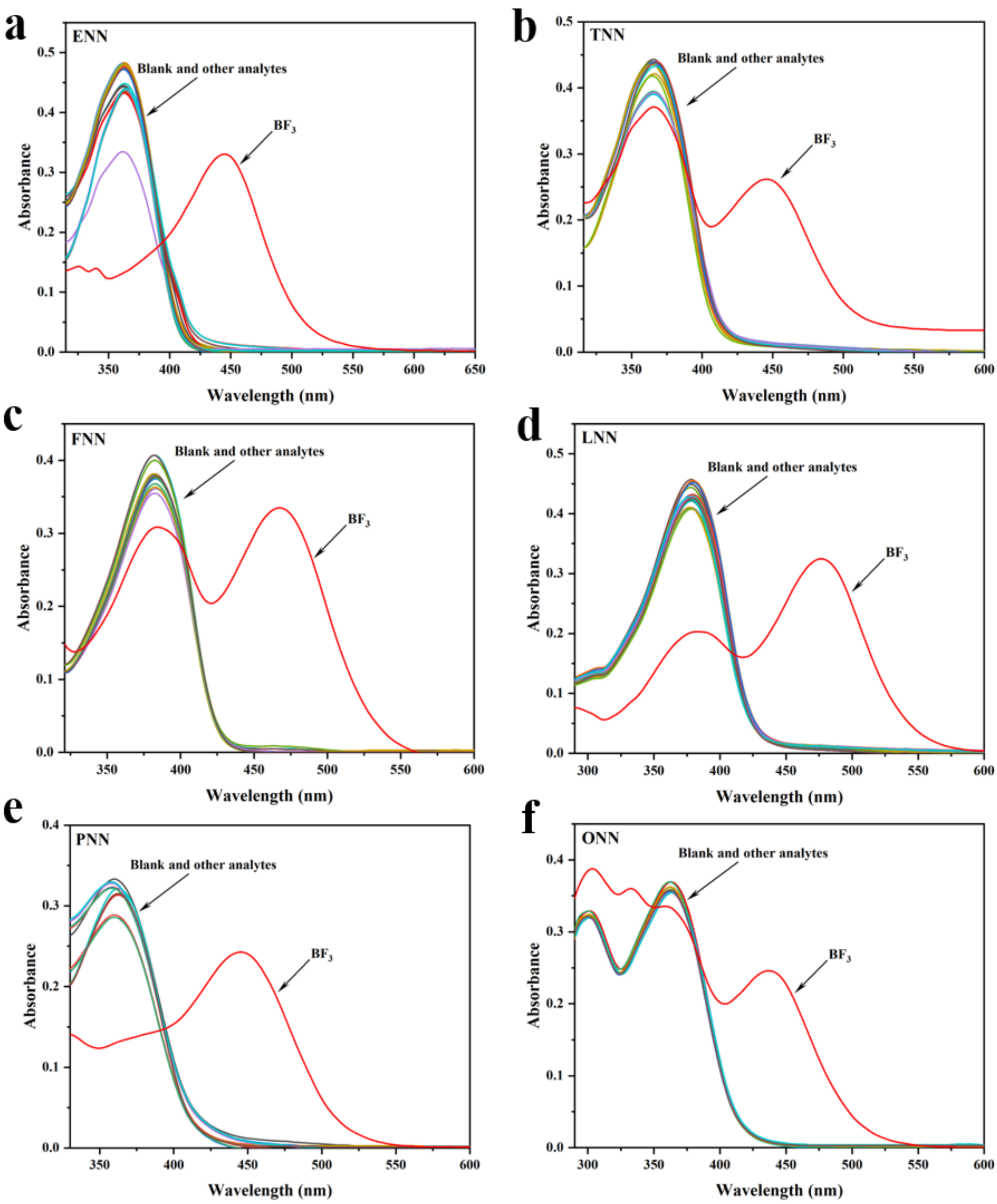

Figure S25. (a–f) UV-vis absorption spectra of sensors HLs with various metal ions and boron derivatives in THF: H_2O (9:1, v/v) solution.

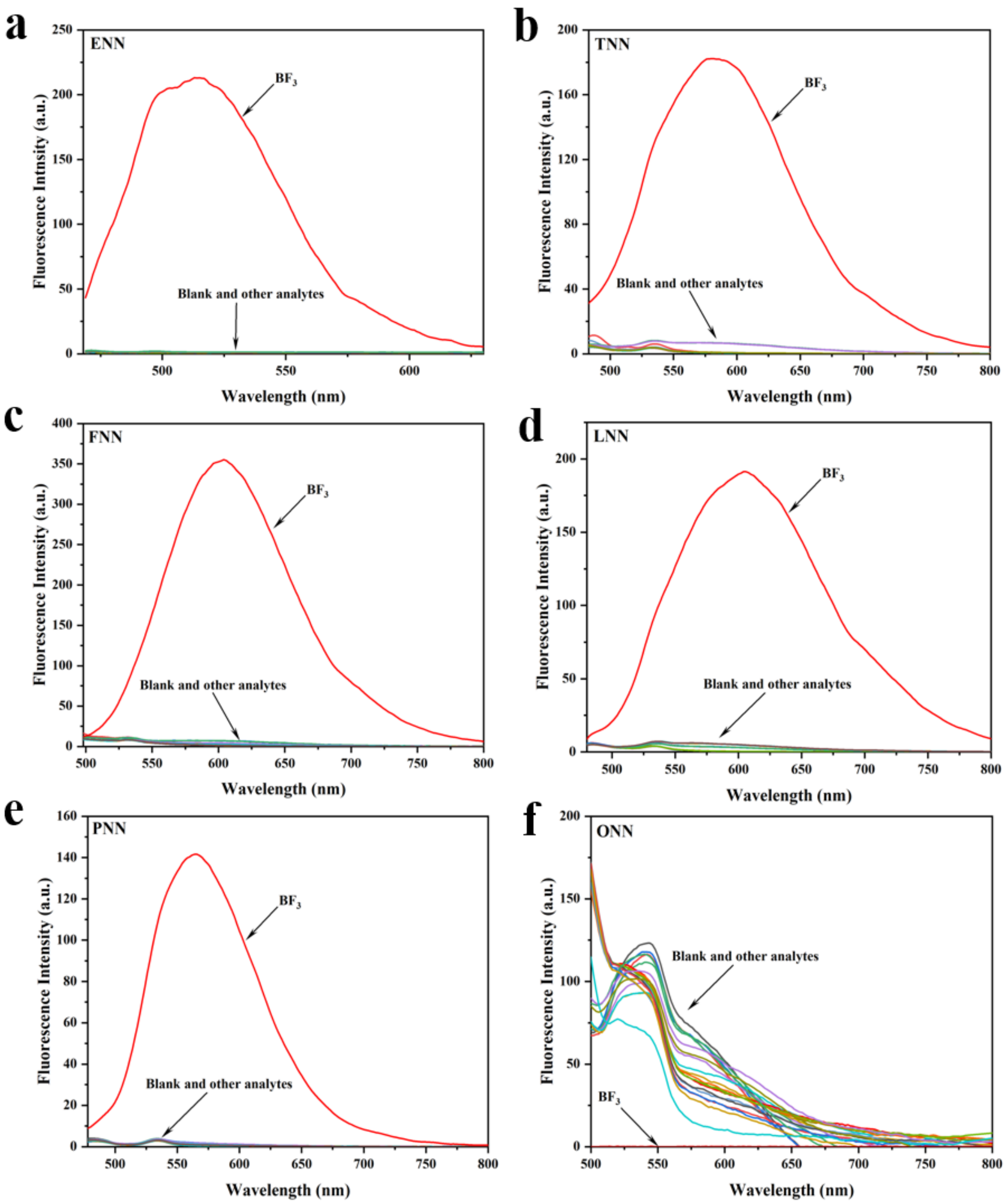

Figure S26. (a–f) Fluorescence (right) spectra of sensors HLs with various metal ions and boron derivatives in THF: H_2O (9:1, v/v) solution.

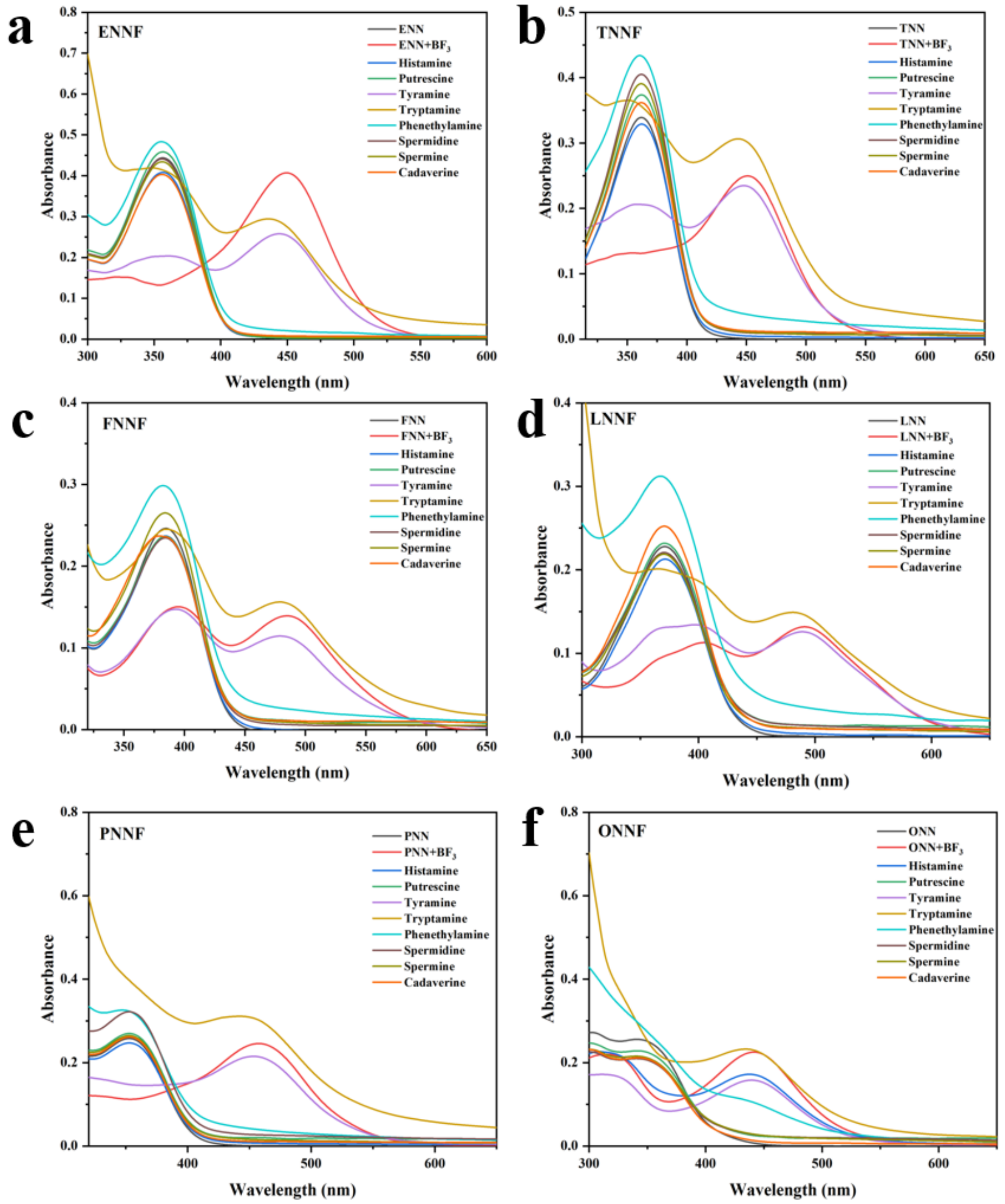

Figure S27. (a–f) UV-Vis spectra of HLs (5 μM) + BF_3 (1equiv) followed by addition of 8 BAs (10equiv) in CH_2Cl_2.

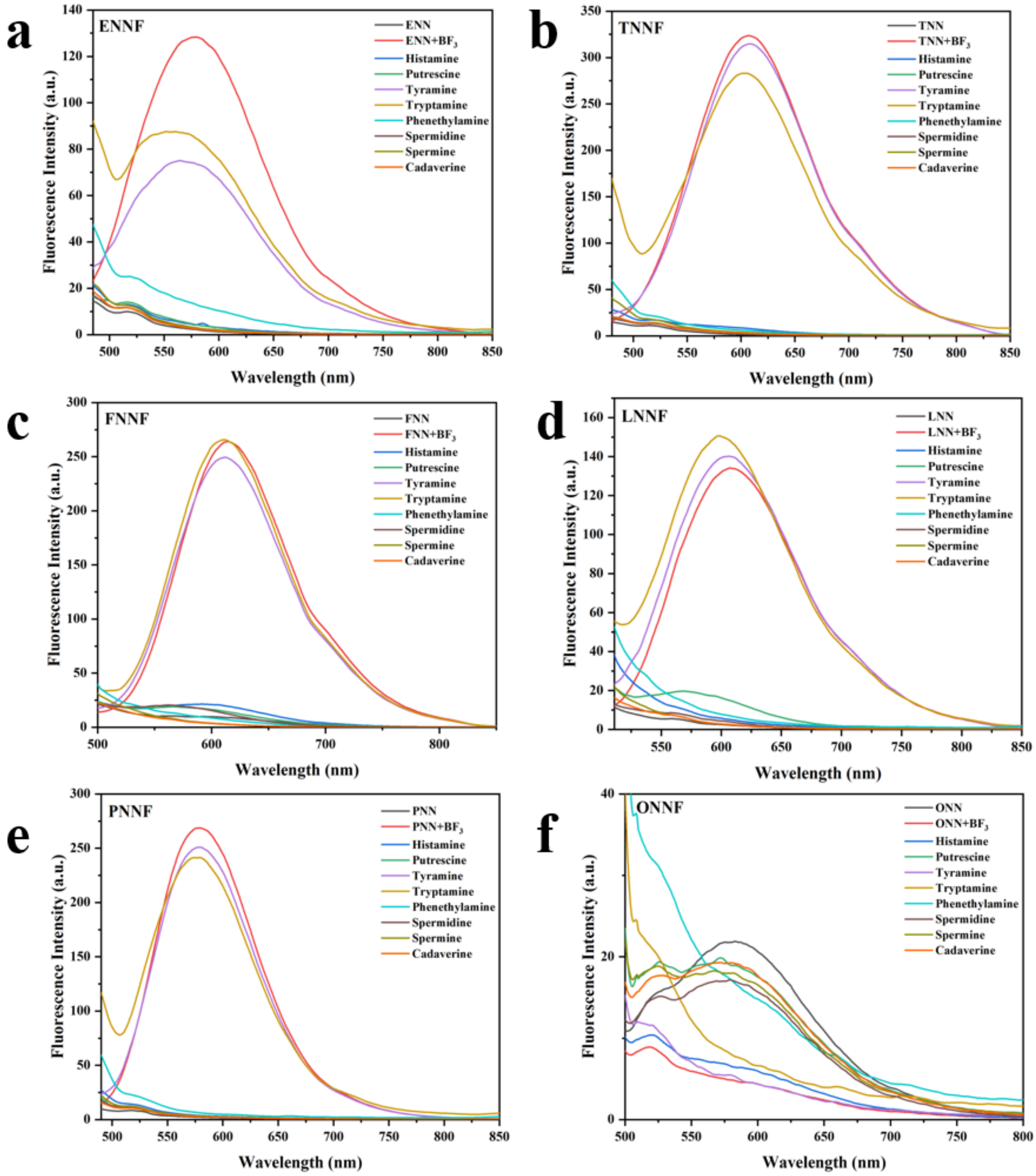

Figure S28. (a–f) Fluorescence spectra of HLs (5 μM) + BF_3 (1equiv) followed by addition of 8 BAs (10equiv) in CH_2Cl_2. The excitation wavelength was 460nm.
